# Subcritical Water Extraction of Kānuka (*Kunzea ericoides*): A Qualitative Analysis of Bioactive Profile Based on Antioxidant Properties

**DOI:** 10.1007/s12033-025-01399-4

**Published:** 2025-02-24

**Authors:** Indhuja Devadass, Simon Swift, Sree Sreebhavan, Saeid Baroutian

**Affiliations:** 1 Department of Chemical and Materials Engineering, The University of Auckland, Auckland, 1010 New Zealand; 2 School of Medical Sciences, Faculty of Medical and Health Sciences, The University of Auckland, Auckland, 1010 New Zealand; 3 Auckland Cancer Society Research Centre, The University of Auckland, Auckland, 1010 New Zealand; 4 Circular Innovations (CIRCUIT) Research Centre, The University of Auckland, Auckland, 1010 New Zealand

**Keywords:** Kānuka, Green extraction, Antioxidant capacity, Polyphenols, Flavonoids

## Abstract

Kānuka (*Kunzea ericoides*) has been traditionally recognised for its health-promoting properties. However, limited comprehensive data on its bioactive profile hinders its potential industrial applications. Therefore, this study investigated the bioactive constituents of the kānuka subcritical water extract, exhibiting maximum antioxidant potential, using LC–MS/MS QTOF. Experiments were performed at different extraction temperatures and times to determine antioxidant content and capacity. The data were statistically analysed to select extracts with the maximum antioxidant response for bioactive screening. The highest recovery of polyphenolics and flavonoids occurred at 170 °C, 5 min (319.96 ± 22.67 mg GAE/g dw and 163.57 ± 9.7 mg QE/g dw), correlating with increased antioxidant activity (DPPH: 87.77 ± 1.74%; ABTS: 97.62 ± 0.12; FRAP: 166.43 ± 25.54 mg TE/g dw). Extracts obtained at 170 °C, 180 °C, 200 °C, and 220 °C with shorter extraction times (5, 10, and 15 min) were selected for bioactive screening using multivariate principal component analysis. Consequently, a rich composition of phenylpropanoids, including flavonoids, phenolic acids, stilbenes, and monolignols, was identified in the kānuka extracts using LC–MS/MS QTOF. Additionally, bioactives previously not found in kānuka extracts were identified, including 2ʹ,6ʹ-Dihydroxy-4ʹ-methoxydihydrochalcone, resveratrol, scopoletin, and naringin, underscoring its potential as a source of valuable metabolites to the pharmaceutical and food industries.

**Supplementary Information:**

The online version contains supplementary material available at 10.1007/s12033-025-01399-4.

## Introduction

Plants are inextricably linked to human lives, providing nutritional and therapeutic needs with their unique phytochemical composition [1]. The therapeutic potential of medicinal and aromatic plants has long been recognised, with their bioactive metabolites playing a crucial role in phytotherapy [2]. These metabolites are produced to defend against biotic and abiotic stressors and are categorised into distinct groups, including phenolics, terpenoids, and alkaloids, based on their specific biosynthetic pathway [3]. The bioactive composition of these metabolites determines the biological properties of the extract. For example, the bioactive profile of *Citrus bergamia* essential oil (linalyl acetate, linalool, and cyclohexane) exhibits antibacterial activity via disrupting cell membrane integrity, enhancing membrane permeability, which leads to leakage of intracellular components and pathogen mortality [4]. Laboratory studies exploring the biological properties of these metabolites have revealed their potential for developing naturally derived metabolites for different industrial applications, including pharmaceuticals and food [1, 5]. Furthermore, the recent advancement in the integration of green syntheses, such as nanoparticle production, and the sustainable extraction methodology (subcritical water extraction and enzyme-assisted extraction) enhances the biological activities of plant-based bioactive compounds [6–9]. This improvement supports their therapeutical potential in various biomedical applications, including antioxidant, antibacterial, anti-inflammatory, and anti-diabetic properties. While numerous medicinal plants have been shown to have a variety of bioactives, only a few of these have been considered safe for human consumption. This limitation is due to the deficit of appropriate and recognised research techniques to access traditional medicine in the laboratory scale screening [10]. As a result, the obtained data is often inadequate to satisfy the healthcare requirement for global acceptance [11]. Consequently, the search for new metabolites that exhibit improved biological response, bioavailability, and safety persists.

**Fig. 1:**
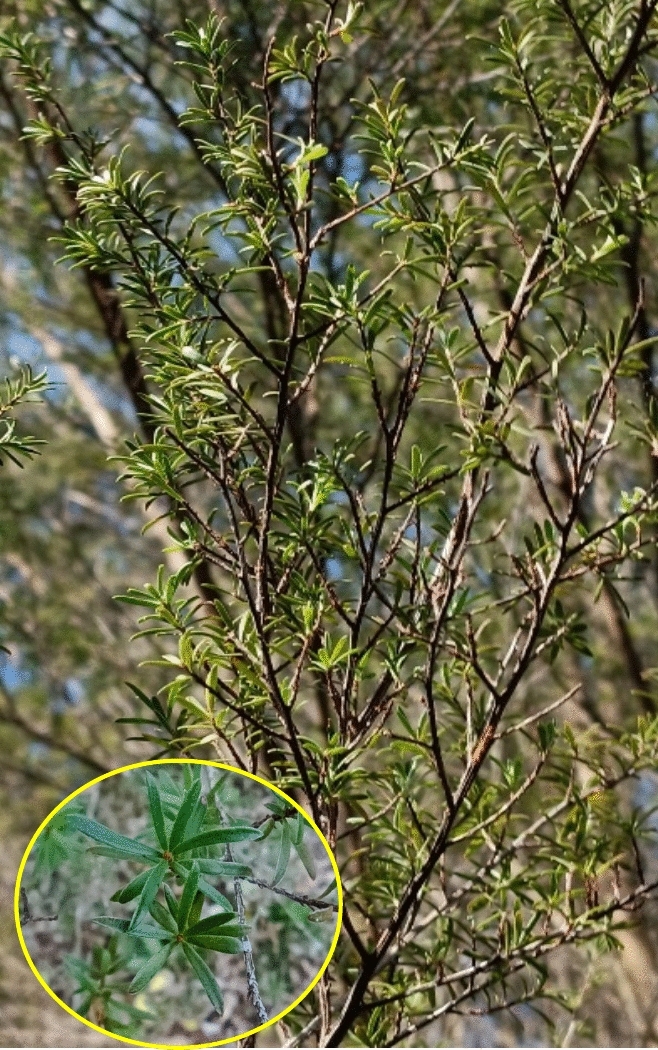
Kānuka (*Kunzea ericoides*) zoomed-in view of its leaves pictured at Auckland Botanical Gardens, Auckland, New Zealand

*Kunzea ericoides* (A. Rich.) Joy Thompson, a member of the Myrtaceae family, is a traditional medicinal plant of New Zealand known as a kānuka or tea-tree plant (Fig. 1). Māori, the indigenous people of New Zealand, have extensive knowledge of using their native plants for spiritual, cultural, and healing purposes. They regarded kānuka as taonga (a treasure), where most plant parts have been utilised for their medicinal value. The decoction of the leaves was consumed to treat a wide range of ailments, including blood disorders, dysentery, common cold, sinus infection, and hay fever, and to support kidney function [12–15]. Bark infusions have been utilised to alleviate fever, induce sleep, and treat oral, throat, and eye troubles [16, 17]. The infusion was applied externally to relieve back pain, while the tree exudate (pia) has been used to soothe burns, scalds and coughing [18, 19] and to treat constipation in children. Apart from its medicinal use, it has been frequently used to prepare beverages [14] and fragrance oil [20]. Beyond its historical medicinal value, kānuka continues to hold relevance in contemporary applications. Its essential oil is commercially preferred to treat dermatophytes, making it a promising candidate for cosmetic products, as an active ingredient in the formulation of disinfectants and relaxants in aromatherapy, supporting respiratory health [21–23].

Recent in vitro biological studies on kānuka leaf extracts obtained with different solvents (ethanol, methanol, and dichloromethane), distillation (steam and hydro), and green extraction (subcritical water extraction and ultrasound-assisted extraction) have demonstrated antioxidant, antimicrobial, anti-inflammatory, spasmogenic and spasmolytic properties associated with the phenolic acids, flavonoids, terpenes, and lipids bioactives [9, 21–27]. Additionally, these studies have demonstrated the influence of extraction in determining the characteristics of the extract, including biological properties, consistency, and fragrance. For instance, recent investigations using ultrasound-assisted extraction have shown improved extract efficiency in inhibiting the enzyme (β-secretase) associated with Alzheimer's disease (AD) compared to solvent extraction methods [26]. Although numerous studies have highlighted kānuka as a source of bioactive metabolites, the observed biological efficiency is less compared to the activity of functional analogues (synthetic compounds) [27–30]. These differences suggest the potential influence of minor compounds in determining the overall biological activities of the extract. Hence, a comprehensive understanding of the metabolic profile of kānuka extract is necessary to elucidate the biological pathway, characterise its interaction with the target, compare its efficiency with the crude extract and assess its contribution to cytotoxicity.

Several studies have quantified the polyphenolic compounds of kānuka using conventional extraction methods, such as solvent and distillation. However, none of these studies have provided compelling evidence to demonstrate its contribution to antioxidant properties due to the limited selectivity of the solvents. Consequently, until 2020, the kānuka were not regarded as a source of antioxidants. In 2020, Essien et al. [9] employed subcritical water extraction (SWE) (150–210 °C) in kānuka leaf. SWE is a sustainable recovery method that uses the thermodynamic property of water at temperatures between 100 and 374 °C and pressures sufficient to maintain the liquid state. At ambient temperature (25 °C), the electrostatic interactions within the water molecules are strong, making them highly polar. As temperature increases, the thermal energy disrupts hydrogen bonding and causes reduced surface tension and viscosity and enhanced solubility and diffusibility of the water [31–33]. Hence, SWE enables the recovery of bioactives and offers greater selectivity and sustainability compared to other conventional extraction methods [33]. This approach identified several polyphenolic compounds (quercetin, chlorogenic acid, catechin, syringic acid, gallic acid, 2-hydroxycinnamic acid, and trans-ferulic acid) with enhanced biological properties, including antioxidant, antibacterial, and antiproliferative [9, 25]. This study presents a promising opportunity to identify novel therapeutic metabolites in kānuka, highlighting it as a valuable source of bioactive compounds.

Identifying bioactive compounds in natural products is challenging due to their structural diversity, including phenolic acids, terpenoids, flavonoids, fatty acids, and alkaloids. Quantifying these metabolites using standard chemicals typically provides information on major compounds, while minor compounds are often neglected. Considering the biological activities of medicinal plants, several studies have reported that the contribution of minor compounds is equally important to the observed biological effects [34, 35]. Hence, qualitative analysis is required to provide comprehensive information on bioactive profiles, which can be achieved using advanced analytical tools, such as liquid chromatography-mass spectrometry-quadrupole time of flight mass spectrometry (LC–MS/MS QTOF). It is used to identify a wide range of natural products by ionising the molecules and separating them based on the mass-to-charge (m/z) ratio as they pass through the electric or magnetic field of the analyser [36].

The current study investigated the bioactive profile of kānuka leaf subcritical water extract, which exhibited maximum antioxidant activity, using LC–MS/MS QTOF. To achieve this, kānuka leaves were subjected to subcritical water extraction at varying conditions. The extraction was performed at different temperatures (60 °C, 80 °C, 100 °C, 120 °C, 140 °C, 160 °C, 170 °C, 180 °C, 200 °C, and 220 °C) and at different time intervals (5–30 min). The antioxidant content and capacity of the extracts were analysed using various assays, including Folin-Ciocalteu, aluminium chloride, 2,2-diphenyl-1-picrylhydrazyl assay, ferric ion-reducing power, and 2,2ʹ-Azinobis-(3-ethylbenzthiazolin-6-sulfonic acid). This approach was designed to explore the distribution of antioxidant molecules below and at subcritical water temperatures. The data was then analysed using principal component analysis (PCA) to observe the correlations between the antioxidant content and capacity. Using this strategy, the samples with maximum antioxidant potential were selected for metabolite screening. The compounds were identified using LC–MS/MS-QTOF equipped with electrospray ionisation (ESI) in both positive and negative ionisation mode. This analysis led to the identification of a novel bioactive compounds, thereby expanding the understanding of kānuka’s phytochemical profile.

## Materials and Methods

### Materials

Kānuka leaves were obtained from Whareponga Valley on the East coast of the North Island of Aotearoa, New Zealand (kindly donated by Nuka Charitable Trust). The species identification was based on the botanical expertise of the source, Nuka Charitable Trust, which is well-established and known for its local knowledge and accurate identification of native plant species. Chemical standards and reagents, including gallic acid, Folin–Ciocalteau reagent, quercetin, aluminium chloride, sodium acetate, sodium nitrate, sodium hydroxide, sodium carbonate, DPPH (2,2-diphenyl-1-picrylhydrazyl), ABTS (2,2′-Azinobis-(3-ethylbenzthiazolin-6-sulfonic) acid), methanol, potassium persulfate, Trolox (( ±)−6-Hydroxy-2,5,7,8-tetramethylchromane-2-carboxylic acid), TPTZ (2,4,6-Tri(2-pyridyl)-s-triazine), ferric chloride, and acetonitrile were purchased from Merck (New Zealand). Nitrogen gas used to pressurise the reactor was purchased from BOC Limited (Auckland, New Zealand).

### Sample Preparation

Kānuka leaves were dried at room temperature. The dried leaves were separated from twigs and ground to a particle size of ≤ 850 µm using an electrical mortar and sieve to increase the surface contact area with the solvent during extraction. The powdered samples were transferred to an air-tight bag and stored in the refrigerator at 4 °C. The extraction was carried out within 2 weeks of sample preparation to ensure minimal degradation of the bioactive compound and to maintain the integrity of the powered sample.

### Subcritical Water Extraction

Subcritical water extraction of kānuka bioactive compounds was performed in a one Litre autoclave batch reactor (Amar Equipment Ltd, India). The reactor was equipped with a PID (proportional integral derivative) controller to regulate temperature, pressure, agitation rate, and a liquid sampling system. The powdered kānuka sample was mixed with the deionised water at a ratio of 15 g/L and transferred to the reactor vessel. The final sample mixture volume was maintained at 600 mL, adhering to the maximum allowable capacity of the reactor for the required temperature. The reactor pressure was maintained at 40 bar, and the samples were agitated at 500 rpm (rotation per minute) during the reaction. Optimum parameters, including solid-solvent ratio, agitation and pressure, were obtained from Essien et al. [9]. Briefly, the sample mixture was purged with nitrogen to remove accumulated oxygen and prevent the oxidation of compounds during extraction. Extraction was performed at ten different temperatures, including 60 °C, 80 °C, 100 °C, 120 °C, 140 °C, 160 °C, 170 °C, 180 °C, 200 °C, and 220 °C. Samples were collected via the sampling port once the reactor reached its set temperature and at 5-min intervals for up to 30 min. Processed samples were vacuum filtered using Whatman® no 1 filter paper (11 µm particle retention size) and stored at – 18 °C for further analyses. A control sample was prepared by incubating grounded kānuka leaves (15 g/L) with deionised water at room temperature for 30 min. After incubation, the sample was filtered and stored similarly to the subcritical water extract.

### pH

The acidity and basicity of extracts were quantified using a pH probe connected to a calibrated pH meter (Hanna edge™, Hanna Instruments, USA).

### Antioxidant Content and Capacity Analyses

Before analyses, the extracts were thawed at room temperature and diluted ten-fold with deionised water. All the assays were performed in a 96-well microplate.

#### Total Polyphenolic Content

The total polyphenolic content of the kānuka extracts was determined using the Folin–Ciocalteu method [37–39] with slight modifications. Folin–Ciocalteu reagent (hetero-poly-phosphotunstate/molybdate, pH 3) was diluted (1:10) with deionised water. 20 µL of diluted kānuka extract was mixed with 100 µL of Folin–Ciocalteu reagent in a 96-well plate and incubated at room temperature for 5 min. After incubation, 100 µL sodium carbonate solution (0.5 M) was added to the reaction mixture. Sodium carbonate was chosen to provide mild alkalinity to the reaction medium, enhancing the reaction rate, minimising interference from reducing sugar, and yielding more reliable results with increased sensitivity compared to other reagents, like sodium hydroxide [40]. The addition of sodium carbonate increases the pH of the reaction mixture to approximately 10, facilitating the dissociation of phenolic protons to form phenolate ions, which are capable of reducing the Folin–Ciocalteu reagent [41]. The reactants in the plate were mixed thoroughly using a micro-plate shaker for two minutes and then incubated in the dark at room temperature for 1 h. The ability of phenolate ion in the sample to reduce Folin–Ciocalteu reagent was measured spectrophotometrically (PerkinElmer 2300 EnSpire Multimode Reader, USA) at 750 nm. The total phenolic content in the extracts was evaluated against the gallic acid standard (0.5–0.03 mg/mL). Results were expressed in milligrams of gallic acid equivalent per gram of the sample's dry weight (mg GAE/g dw).

#### Total Flavonoid Content

The total flavonoid content of the kānuka extracts was determined using the aluminium chloride (AlCl_3_) method as described by Essien et al. [9]. 25 µL of diluted kānuka extract was added to 100 µL of distilled water. 10 µL of 5% sodium nitrate solution was added to the diluted extract and incubated for 5 min at room temperature. After incubation, 15 µL of 10% AlCl_3_ solution was added to the reaction mixture and incubated for 6 min at room temperature. Finally, 50 µL of 1 M sodium hydroxide solution was added and diluted with 50 µL distilled water. The reaction mixture was incubated in the dark for 1 h. The absorbance was measured at 510 nm using a microplate spectrophotometer and quantified against the quercetin standard (ranging from 0.5 to 0.03 mg/mL). Results were expressed in milligram quercetin equivalent per gram of the sample's dry weight (mg QE/g dw).

#### 2,2-Diphenyl-1-picrylhydrazyl (DPPH) activity

The DPPH free radical scavenging activity of kānuka extracts was determined using the method described by Herald et al. and Susawaengsup et al. [39, 42] with slight modifications. The required molar concentration of DPPH reagent was evaluated before the analysis. Different millimolar concentrations of DPPH (0.15 mmol/L, 0.2 mmol/L, 0.25 mmol/L, 0.3 mmol/L, and 0.35 mmol/L) were prepared with 70% methanol and standardised using Trolox at concentrations ranging from 0.5 mg/mL to 0.03125 mg/mL. A linear correlation coefficient *R*^2^ = 0.9961 obtained with the 0.35 mmol/L DPPH was selected for subsequent analysis.

20 µL of diluted kānuka extracts were mixed with 180 µL of 0.35 mmol/L DPPH reagent dissolved in 70% methanol. The mixture was then incubated in the dark at room temperature for 30 min. The reduction potential of the extracts was measured spectrophotometrically at 517 nm and quantified against the Trolox standard curve. Results were expressed as milligram Trolox equivalent antioxidant capacity per gram of dry weight (mg TEAC/g dw). A control sample without kānuka extract was used to measure baseline DPPH absorbance. The percentage of radical scavenging activity (RSA) was calculated using Eq. 1.

1 $$\% {\mathrm{of}}\;{\mathrm{RSA}} = \frac{{\left( {A_{{{\mathrm{control}}}} - A_{{{\mathrm{sample}}}} } \right)}}{{A_{{{\mathrm{control}}}} }} \times 100$$

where *A*_control_ and *A*_sample_ are the absorbances of the DPPH reagent without and with kānuka extract, respectively.

#### 2,2ʹ-Azinobis-(3-ethylbenzthiazolin-6-sulfonic acid) (ABTS) Activity

The ABTS^●+^ radical reduction potential of kānuka extracts was determined using a method described by Re et al. [43] with slight modification. A 7 mM concentration of ABTS reagent was dissolved in sodium acetate buffer (20 mM, pH 4.5). The oxidation of the ABTS solution was initiated by adding 2.45 mM potassium persulfate at a 1:1 (v/v) ratio and incubating for 12 to 16 h. The absorbance of the oxidised reagent mixture (ABTS^●+^) was adjusted to 1.6 ± 0.02 at 734 nm with 70% methanol.

The assay was performed by adding 180 µL of ABTS^●+^ (blue-green chromophore) reagent with 20 µL of diluted kānuka extracts. The reaction mixture was then incubated for 2 to 5 min at room temperature. The reduction capacity of kānuka extracts was measured spectrophotometrically at 734 nm and quantified against Trolox (dissolved in 70% ethanol) standards ranging from 0.5 to 0.03 mg/mL. The results were expressed as milligram Trolox equivalent antioxidant capacity per gram of dry weight (mg TEAC/g dw). The percentage of the ABTS^●+^ radical reduction was calculated using Eq. 2.

2 $${\mathrm{of}}\;{\mathrm{ABTS}}\;{\mathrm{radical}}\;{\mathrm{reduction}} = \frac{{\left( {A_{{{\mathrm{control}}}} - A_{{{\mathrm{sample}}}} } \right)}}{{A_{{{\mathrm{control}}}} }} \times 100$$

where *A*_control_ is the absorbance of the ABTS^●+^ reagent, and *A*_sample_ is the absorbance of samples with the ABTS^●+^.

#### Ferric Ion-Reducing Antioxidant Power (FRAP)

The Ferric complex (FRAP reagent) was prepared by mixing 20 mmol/L FeCl_3_ in a 10 mmol/L TPTZ solution and 300 mmol/L sodium acetate buffer (pH 3.6) in a 1:1:10 v/v/v ratio. 10 µL of kānuka extracts was added to the 200 µL FRAP reagent and incubated for 10 min at room temperature. The formation of ferrous ions (FE^II^-TPTZ) was measured spectrophotometrically at 593 nm. The ferric ion-reducing potential of the samples was quantified using a Trolox (dissolved in 70% ethanol) standard curve ranging from 0.5 to 0.03 mg/mL. A reagent without a sample served as a control. Results were expressed in milligrams of Trolox equivalent antioxidant capacity per gram of the sample's dry weight (mg TEAC/g dw) [9].

### Statistical Analysis

All experiments were performed in duplicates to enhance the reproducibility of the results and minimise potential experimental variability. Mean and standard deviations were calculated using Microsoft Excel to understand the data distribution and central tendency. One-way ANOVA was performed to study significant differences between variables (temperature and time) and understand the statistical significance of experimental variations. The relationship between antioxidant content and capacity was examined using Pearson's correlation in SPSS with a significant *p* < 0.01 (two-tailed) to ensure high confidence in the correlation analysis. Principal component analysis was performed using OringinPro 10.10.170 software to visualise significant variations within the data and select the appropriate extract for subsequent bioactive screening. The bioactive screening for each sample was performed in triplicates.

### LC–MS/MS QTOF Analysis

Non-volatile and polar components of the kānuka extracts were identified using LC–MS/MS QTOF 6530 (Agilent Technologies, Santa Clara, CA, USA) equipped with Dual Agilent Jet Stream electrospray ionisation. The screening method was developed for kānuka extracts using the reference from Kumar et al. [44] with a few modifications.

50 µL of the undiluted kānuka extract was diluted with 50 µL of diluent consisting of 50% acetonitrile (ACN) in water with 0.1% Formic acid (50% acetonitrile, 0.1% formic acid, and 0.1% Milli-Q water). Diluted sample (25 µL) was injected to pass through the reverse phase chromatographic column, Zorbax SB-C18 (5 µm particle size, 3 mm diameter, and 150 mm length) (Agilent Technologies, Santa Clara, CA, USA) at the flow rate of 0.4 mL/min. The autosampler temperature was maintained at 8 °C, and the column temperature was set to 40 °C. Components were separated using eluting solvent A, containing 0.1% formic acid in Milli-Q water and solvent B, 100% acetonitrile with 0.1% formic acid. The elution gradient was programmed as follows: 0–10 min, 20% B; 10–15 min, 40% B; 15–25 min, 60% B, 25–30 min, 90% B; 30–35 min, 20% B.

The analyte in solvent was converted to gas phase ions through desolvation, where the solvent was evaporated by sheath (nitrogen) gas at 350 °C, flow rate 10 L/min, and droplet fission. Compounds were identified in both positive and negative ionisation modes. Charged particles were directed towards the mass spectrometry to separate the molecules according to the mass-to-charge ratio (m/z). Mass scanning range was set at 50–2000 m/z for MS and 50–1500 m/z for MS/MS. The collision energy of 15 eV was applied to fragment the parent ions into daughter ions to reveal molecular characteristics. Other mass spectrometry parameters included a fragmentor of 220 V, capillary voltage of 4000 V, nebuliser pressure of 40 psi, and nozzle voltage of 500 V. The method was validated using reference peak (Hexakis(2,2,3,3-tetrafluoropropoxy)phosphazene (C_18_H_18_F_24_N_3_O_6_P_3_), molecular mass- 921.00252, in positive mode-922.0098) has been used during the method development and removed during the compound identification to avoid interference with the other compounds.

### Compound Identification and Data Analysis

Data were processed using Agilent MassHunter Qualitative analysis software B.10.0. Compounds were identified using the Personal Compound Database Library (PCDL), containing molecular formula, molecular mass, and compound name sourced from literature and Kyoto Encyclopaedia for Gene and Genome Pathways (KEGG—phytochemical compounds) database. Compounds with an accuracy of ± 1 to 5 ppm and high mass resolution (> 10^3^) were accepted. Product ion of selected compounds was further confirmed by comparing spectra available in different public networks, including MassBank (REKIN_ReSpect), NIST, PubChem, and ChemSpider.

## Results and Discussion

### Effect of Time and Temperature on the pH of Kānuka Extracts

**Fig. 2:**
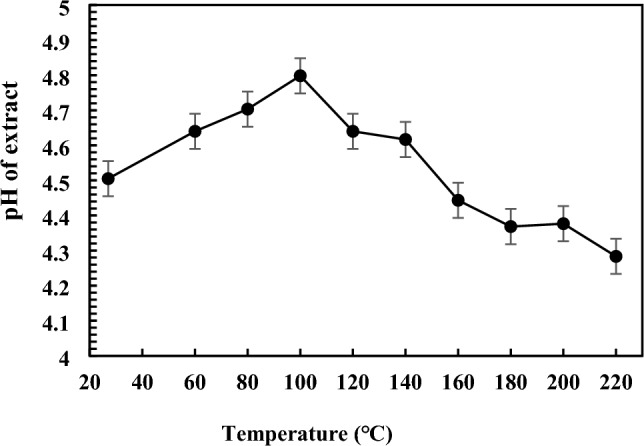
Effect of temperature (25 °C, 60 °C to 220 °C) on the pH of kānuka subcritical water extract. Each point is the mean pH of the extract (*n* = 2), with error bars representing the standard deviation

The hydrogen ion concentration (pH) is an important factor in plant extracts, predominantly used in the food, beverage, and pharmaceutical industries concerned with consumer safety [45]. The pH has been demonstrated to impact the biological efficiency of the extract, such as antioxidant activity [46, 47]. Factors influencing the pH of the extract may include extraction temperature, type of solvent, and phytochemical composition. The mean and standard deviation of the pH of kānuka extracts obtained by SWE under different conditions, as well as control (25 °C), are presented in Fig. 2. Temperature significantly influenced the extract's pH (*p* ≤ 0.05), while extraction time did not. The overall pH variations in the extract ranged from 4.3 to 4.8.

The pH of kānuka extracts gradually increased to 4.8 when heated from 60 to 100 °C, indicating the self-ionisation of water, defined as the dissociation of water molecules (H_2_O) into hydronium ion (H_3_O^+^) and hydroxide ion (OH^−^), impacting pH variation. At lower temperatures, the dissociation constant is higher (25 °C: 1.0 × 10^−14^; 100 °C: 5.6 × 10^−13^), resulting in an increased concentration of OH^−^ ion and a consequent rise in pH. Conversely, as temperature increases, the concentration of H_3_O^+^ ions increases, which lowers the pH of the extract [32]. The pH of the kānuka water extract (control) is 4.5, which is acidic.

The acidic pH of the extract plays a crucial role in maintaining the stability and activity of phenolic compounds, preventing degradation and preserving their bioactive properties [48]. Acidic conditions are particularly favourable for maintaining the stability of phenolic compounds, such as caffeic acid, gallic acid, and chlorogenic acid, which undergo potential degradation with irreversible spectral transformations in alkaline conditions (pH 9). Conversely, some phenolic compounds, such as (-)-catechin, ferulic acids, and trans-cinnamic acid, were more stable at varying pH 3–11 [49]. Studies have shown that plant aqueous extracts (*Tamarindu indica* Linn.) with lower pH have greater antioxidant activity (pH 6), while acetone extract exhibits higher antimicrobial efficiency (pH 2 to 4) than extracts with alkaline pH 6 to 10 [50]. According to Reddy et al. [45], beverages with a pH range from 4 to 6 are minimally erosive, indicating they are less likely to cause dental problems. Given that the pH of kānuka extracts ranges from 4.8 to 4.3, these extracts fall within the minimally erosive range. This combination of stability, enhanced bioactivity, and safety makes kānuka extract’s pH profile valuable for commercial applications in the food and pharmaceutical industries.

### Antioxidant Content and Capacity

#### Temperature-Dependent Variation Observed in the Polyphenolic Recovery

The maximum total phenolic content (TPC) of kānuka water extracts (control) was observed as 120.17 ± 1.97 mg GAE/g dw. The recovery of TPC gradually increased at elevated temperatures (Fig. 2a). It demonstrates the weakening of hydrogen bonds in water molecules at higher temperatures and controlled pressure, favouring London dispersion forces. These forces make water less polar, enhancing the solubility of non-polar compounds and accelerating the recovery process [32]. The results suggest that temperature had a significant (*p* < 0.05) influence on the recovery of TPC. Although minor variations were observed based on different time intervals, statistical evidence did not support (*p* = 0.569) its influence on antioxidant recovery. However, colour changes in the extracts were observed at increasing times, which is consistent with the observations of Essien et al. [9].

**Fig. 3:**
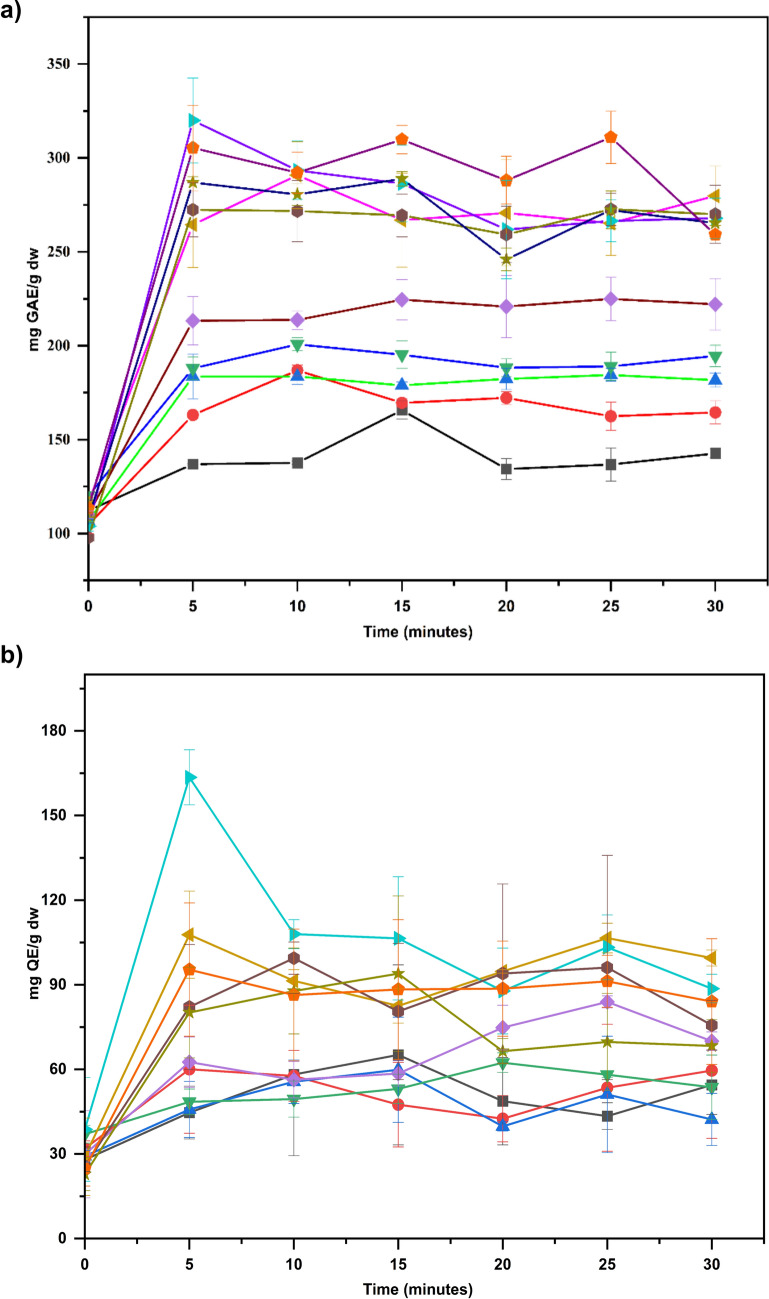
Effect of extraction temperature (black square - 60 °C (black), black circle - 80 °C (red), black triangle - 100 °C (green), black down-pointing triangle −120 °C (blue), black diamond suit - 140 °C (wine), black left-pointing pointer −160 °C (magenta), black right-pointing pointer - 170 °C (violet), black hexagon - 180 °C (dark yellow), star - 200 °C (navy), black pentagon - 220 °C (purple) and time on the recovery of total polyphenolics (a) and flavonoid compounds (b) of kānuka subcritical water extract, where time 0 is the control extract obtained at 25 °C. Each point represents the mean value of TPC (a) and TFC (b) (*n* = 6), with an error bar representing the standard deviation

Additionally, Fig. 3a illustrates a notable variation in the recovery of TPC. Below 140 °C, TPC recovery is significantly (*p* < 0.05) lower than above 140 °C, effectively dividing the data into two distinct groups. However, within these groups, TPC does not exhibit significant differences. It indicates that the critical temperature threshold for the TPC recovery in kānuka is 140 °C. Similarly, the maximum time required for optimal TPC recovery across all tested temperatures was observed to be between 5 and 10 min. These results are consistent with findings by Rangriwong et al. [51], which demonstrated that polyphenolic compounds could be efficiently recovered within 10 min of SWE in *Terminalia chebula* Retz. fruit. Additionally, the recovery of TPC from 20 different medicinal plants (at 20% concentration) using SWE demonstrated that the optimum temperature for maximum TPC recovery was 75 °C [48]. This outcome highlights the efficiency of SWE in recovering TPC in a relatively shorter time, which is advantageous in reducing energy consumption for large-scale applications.

The maximum TPC was obtained at 170 °C for 5 min (319.96 ± 22.67 mg GAE/g dw), which does not correlate with the previous finding where the highest antioxidant activity was observed at 170 °C for 20 min (172.81 ± 1.9 mg GAE/g dw) [9]. This discrepancy highlights the influence of quantification parameters, storage duration, and processing conditions. The substantial 85% increase in quantification could be attributed to the modification in assay parameters in this study, such as altering pH with the different molar concentrations of sodium carbonate. Additionally, the limitations of the Folin–Ciocalteu assay must be considered. Fabian et al. [52] reviewed the disadvantages of using Folin–Ciocalteu reagent for TPC analysis, noting an increased chance of false-positive results due to the reduction of reagent by other metabolites, including protein, amino acids, and carbohydrates, which diminishes the reliability of data reproduction. This outcome indicates that optimising assay parameters is necessary to avoid variation in quantification.

After 170 °C, TPC gradually decreased with increasing temperature. However, at 220 °C (15 to 25 min), a significant increase in TPC recovery (259.21 ± 1.25 mg GAE/g dw) was observed. It demonstrates the complex relationship between temperature and polyphenolic compound recovery. This outcome aligns with the findings of He et al. [53], who reported maximum TPC recovery (48.54 mg/g dw) at 220 °C (30 min) using SWE in pomegranate seed residues. It indicates that the possible breakdown of complex polyphenolic compounds into simple molecules at higher temperatures might have favoured the increased expression of TPC.

In addition, the TPC at lower temperatures (60 °C) is evidence that kānuka inherently contains antioxidant molecules. It indicates that even mild extraction can effectively recover antioxidant molecules in kānuka, reinforcing the traditional Māori practice of consuming kānuka water infusion or decoction as a beverage (tea). Consequently, the outcome of TPC recovery using SWE demonstrates the distribution, stability, and extractability of kānuka polyphenolic compounds at different extraction temperatures.

#### Temperature Modulates Flavonoid Recovery Based on the Functional Group

The maximum flavonoid content (TFC) in kānuka water extract (control) was 36.99 ± 3.7 mg QE/g dw. These findings differ from previous studies in our laboratory, where flavonoids were predominant in extracts of the same batch of leaves (91.52 ± 5.28 mg QE/g dw) [9]. The observed 60% reduction in flavonoid content may have resulted from oxidative degradation during storage. Reduction in antioxidant contents during processing plant matrices and storage is a common phenomenon reported in several studies [54, 55]. For example, Baltacioğlu et al. [56] observed an 86% reduction in flavonoid (quercetin-3-d-galactoside) in rowanberry within 16 days of storage (22 °C) due to enzymatic degradation. In the context of this kinetic study, it can be hypothesised that flavonoid content in kānuka might have decreased over storage. Additionally, Korus et al. [54] demonstrated that blanching significantly reduced the polyphenolic content (32%), vitamin C (15%), and antioxidant reducing capacity (13%) in kale leaves compared to fresh leaves, before subjecting them to storage methods (air-drying and freeze-drying).

Like TPC, extraction temperature significantly influenced the recovery of TFC (Fig. 3b). The maximum recovery of the flavonoids was obtained at 170 °C for 5 min (163.57 ± 9.75 mg QE/g dw). These findings align with the study of Putra et al. [57], who demonstrated maximum TFC recovery in dried peanut skin at temperatures between 120 °C and 160 °C using subcritical water extraction. Using the Chrastil statistical model, the author explained that higher temperatures increase the water’s diffusivity and solvation power, enabling efficient extraction with minimal (average absolute relative deviation of 4.47%) compound degradation. However, no consistent trend in TFC was observed under different extraction conditions in kānuka. For example, although TFC recovery was efficient within 5 min of extraction, a sudden increase at 25 min cannot be neglected. The observed fluctuations explain the varying interactive abilities of flavonoid functional groups with the solvent properties of water in SWE.

Flavonoids are a diverse group of polyphenolic compounds with various functional groups, including hydroxyl (OH), double bond (C_2_–C_3_), methoxylated (O–CH_3_), and sugar molecules (mono- or disaccharides). According to Ko et al. [58], these functional groups are vital in determining the optimum extraction temperature in SWE. For example, flavonoids with OH groups can be extracted at lower temperatures (170 °C/10 min) than those with C_2_–C_3_ and O–CH_3_ groups (190 °C/15 min). Similarly, monosaccharide compounds require lower temperatures (110 °C/15 min) than disaccharides (170 °C/15 min).

Therefore, based on the TFC outcome of kānuka extracts, flavonoids with glycoside compounds might have been recovered in a shorter time (5 min), while the aglycone group required an extended extraction time (25 min). The results indicate that kānuka extracts contain more glycoside flavonoids at 170 °C (5 min) than aglycones. These findings align with the study of Wyatt et al. [27], who observed an eight-fold higher proportion of glycoside flavonoids in kānuka solvent (methanol) extract than aglycone.

#### Free-Radical Scavenging Activity Enhanced with a Higher Proportion of Polar Molecules

**Fig. 4:**
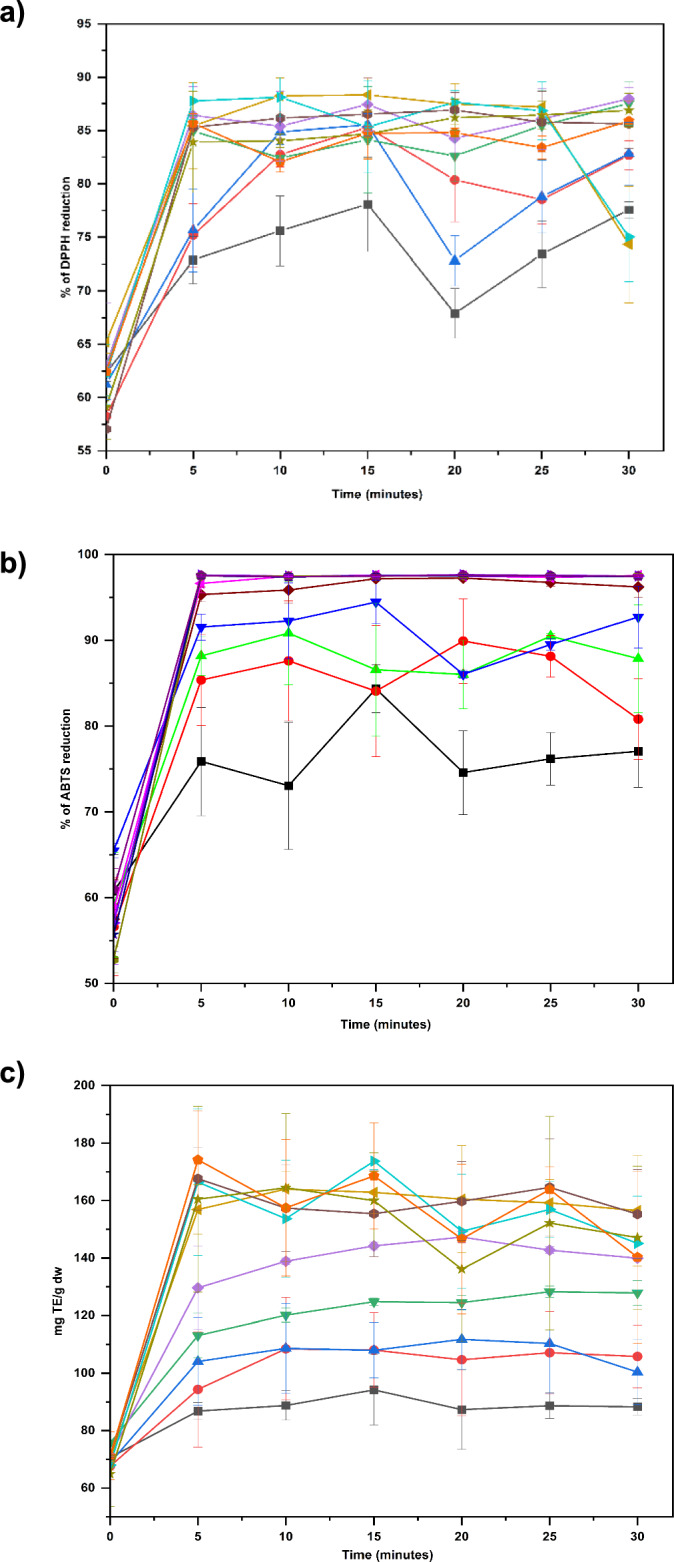
Effect of extraction temperature (black square - 60 °C (black), black diamond suit - 80 °C (red), black triangle - 100 °C (green), black down-pointing triangle −120 °C (blue), black circle - 140 °C (wine), black left-pointing pointer - 160 °C (magenta), black right-pointing pointer - 170 °C (violet), black hexagon −180 °C (dark yellow), star - 200 °C (navy), black pentagon - 220 °C (purple) and time on percentage of free radical scavenging, including (a) DPPH and (b) ABTS and (c) ferric reducing properties (FRAP) of kānuka subcritical water extract, where time 0 is the control extract obtained at 25 °C. Each point represents the mean values of the extract's RSA and ferric ion-reducing activity (*n* = 6), with the error bar representing the standard deviation

The RSA of kānuka water extract (control) was observed as 65.18 ± 5.03% and 65.49 ± 0.87% for DPPH and ABTS^●+^, respectively. This observation highlights the natural antioxidant ability of kānuka, regardless of the extraction method used. As anticipated from TPC and TFC outcomes, the RSA of the kānuka extracts increased gradually with increasing temperatures. The maximum reduction of 88.77 ± 1.74% and 97.5 ± 0.12% were observed at 170 °C (10 min) for DPPH and ABTS, respectively (Fig. 4a, b). It aligns with Majid & Silva's [26] findings, which reported 72.40 ± 2.40% RSA in kānuka extract at 69 °C using ultrasound-assisted extraction.

Above 120 °C, the RSA plateaued between 80 and 90% in both assays. It could be attributed to the saturation effect of kānuka extract beyond the threshold temperature, likely due to equilibrium conditions. In such states, there may not be sufficient free radicals for antioxidant molecules to donate/accept electrons/hydrogen or vice versa. It indicates that optimising assay parameters, including DPPH and ABTS● + molar concentration and extract dilution, is necessary for critically evaluating antioxidant properties.

Comparing both RSA, ABTS exhibited a significantly higher reduction potential than DPPH, a trend commonly observed in the studies associated with plant extracts [59, 60]. This highlights the sensitivity of ABTS^●+^ in detecting higher reaction kinetics in hydrophilic and lipophilic extracts compared to DPPH, which is more sensitive to hydrophobic extracts. Thus, higher ABTS● + inhibition suggests that kānuka extracts may contain more polar antioxidant molecules than non-polar ones.

#### The Ferric Ion-Reducing Potential is Associated with Total Polyphenolic Content

The ability of kānuka extracts to reduce ferric ions to ferrous ions in the presence of a reducing agent TPTZ was investigated and presented in Fig. 4c. The maximum FRAP of kānuka water extract (control) was 75.73 ± 3.85 mg TE/g dw. As temperature increases, the ferric ion-reducing activity of kānuka extracts also increases. The maximum ferric ion reduction was observed at 170 °C for 15 min (173.73 ± 2.96 me TE/g dw). Additionally, a correlation between the results for TPC (Fig. 3a) and FRAP (Fig. 4c) was noted. It indicates that phenolic compounds in kānuka extract exhibit a greater affinity for reducing metal ions than other free radical scavenging activities (ABTS and DPPH), as Essien et al. [9] reported.

The principle of the redox mechanism elucidates the observed increased affinity. According to Yoshino & Murakami [61], both flavonoids and non-flavonoids participate in iron-reducing activity. Flavonoids promote the autooxidation of ferric ions (prooxidants), while non-flavonoids form inactive complexes with polyphenols. Subsequently, the ferric-polyphenol complex prevents free radical formation and lipid peroxidation, reducing oxidative stress. Thus, the metal-chelating properties of antioxidant molecules are highly correlated with TPC.

#### Correlation Between Antioxidant Content and Capacity

The linear relationship (*r*) between antioxidant content (TPC and TFC) and its capacity (DPPH, ABTS, and FRAP) was investigated using Pearson's correlation coefficient with a significant *p*-value of 0.01 (Table 1). TPC and TFC exhibited a strong positive correlation with all antioxidant assays tested, especially with FRAP, with *r* = 0.976 and *r* = 0.874, respectively. The strong relationship between the polyphenolic compounds and the metal-chelating activity has been commonly observed in antioxidant studies in plant extract [62]. A weaker correlation was observed between the TFC and DPPH, with *r* = 0.683. These findings align with the previous study, which reported a lower correlation between TFC and DPPH (*r* = 0.492) and a stronger correlation between TPC and FRAP (*r* = 0.914) in kānuka extracts [9].

**Table 1:** Pearson correlation coefficient (*r*) of antioxidant content and capacity

	TPC	TFC	DPPH	ABTS	FRAP
TPC	1	–	–	–	–
TFC	0.893**	1	–	–	–
DPPH	0.768**	0.683**	1	–	–
ABTS	0.892**	0.764**	0.896**	1	–
FRAP	0.976**	0.874**	0.809**	0.913**	1

**Correlation is significant at the 0.01 level (2-tailed)

Principal component analysis is a statistical multivariate analysis performed to identify key variables, including temperature and time, strongly influencing the recovery of antioxidants and their relationship with corresponding bioactivity (antioxidant capacity). A biplot was plotted to visualise the loading direction of the tested variables (TPC, TFC, DPPH, ABTS, and FRAP) and observation clusters based on the two independent variables.

**Fig. 5:**
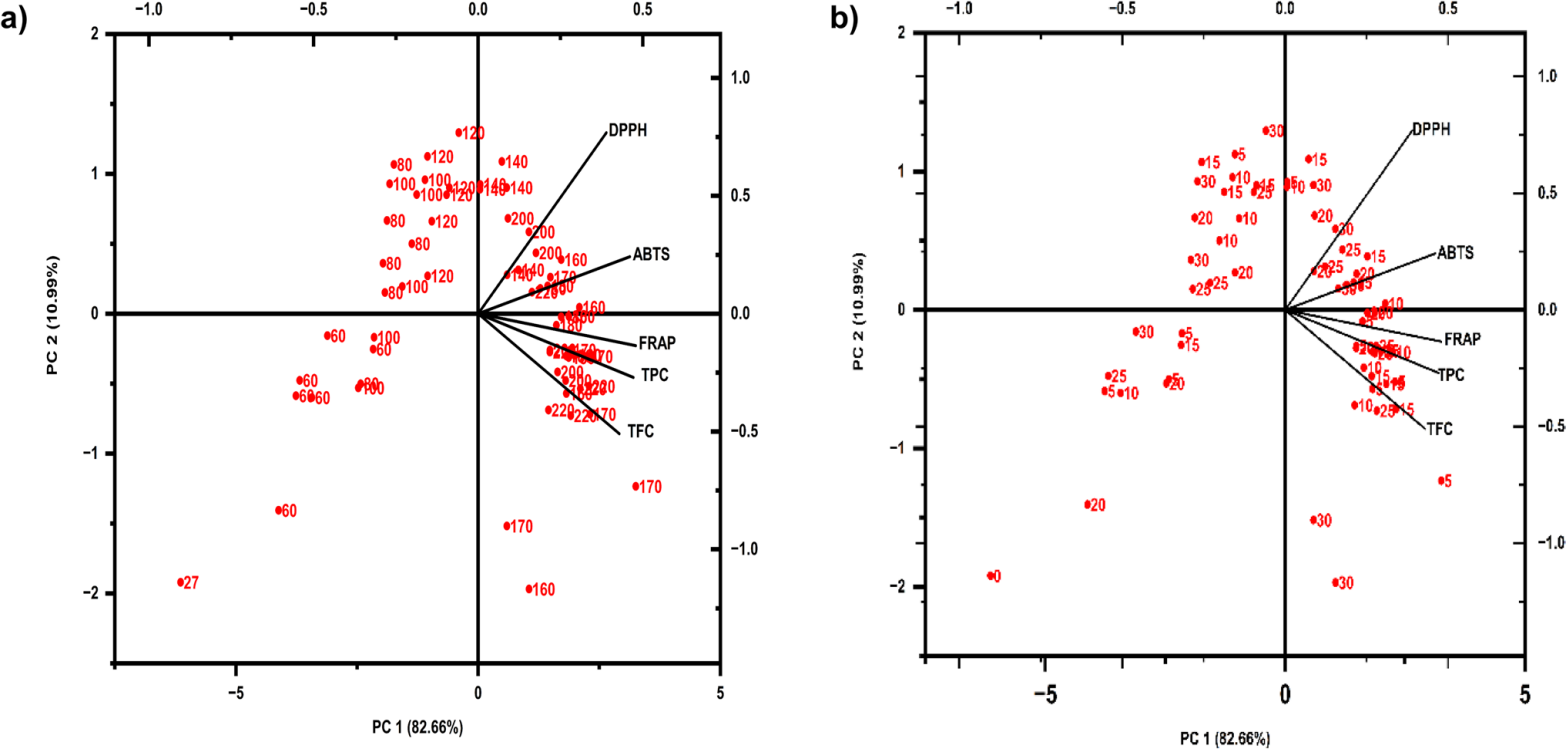
Principal component analysis of antioxidant content and capacity of kānuka subcritical water extract. Biplot based on different temperatures (a) and time (b)

PC1 showed a higher proportion of the dataset (87.88%) than PC2 (7.75%). According to the score values in Fig. 5a, data obtained at temperatures above 160 °C clustered together, indicating a strong influence on antioxidant recovery and activity compared to temperatures below 140 °C. Data points at 120 °C and 140 °C, falling on the PC1 axis, appear to separate the data into two clusters. This observation indicates that the optimal temperature (or minimum threshold) to initiate the kānuka extraction using SWE is either 120 °C or 140 °C. This finding is consistent with the recovery of the TPC assay, highlighting 140 °C as a critical temperature for TPC recovery. Similarly, PCA data based on different times (Fig. 5b) indicate no variations were observed after 15 min of extraction.

The magnitude of the loading plot on each parameter indicates the strength of the relationship between tested variables. It reveals that TPC exhibits a strong positive relationship with antioxidant assays, with the order of correlation strength being FRAP > ABTS > DPPH. Likewise, TFC is weakly correlated with DPPH and ABTS. Based on these statistical observations, the extracts obtained at higher temperatures (170 °C, 180 °C, 200 °C, and 220 °C) and shorter extraction times (5, 10, and 15 min), which exhibited maximum antioxidant content and capacity were selected for the identification of their bioactive profile using LC–MS/MS-QTOF.

### Bioactive Profile of Kānuka Extract

LC–MS/MS QTOF was employed to identify bioactives in kānuka extracts obtained using SWE at various temperatures (Control: 25 °C; 170 °C, 180 °C, 200 °C, and 220 °C) and time points (5, 10, and 15 min). Polyphenolic compounds are synthesised via the phenylpropanoid pathway. Based on their chemical configuration, they are divided into different groups, including flavonoids, monolignols, stilbenes, coumarins, and phenolic acids [63].

**Fig.6:**
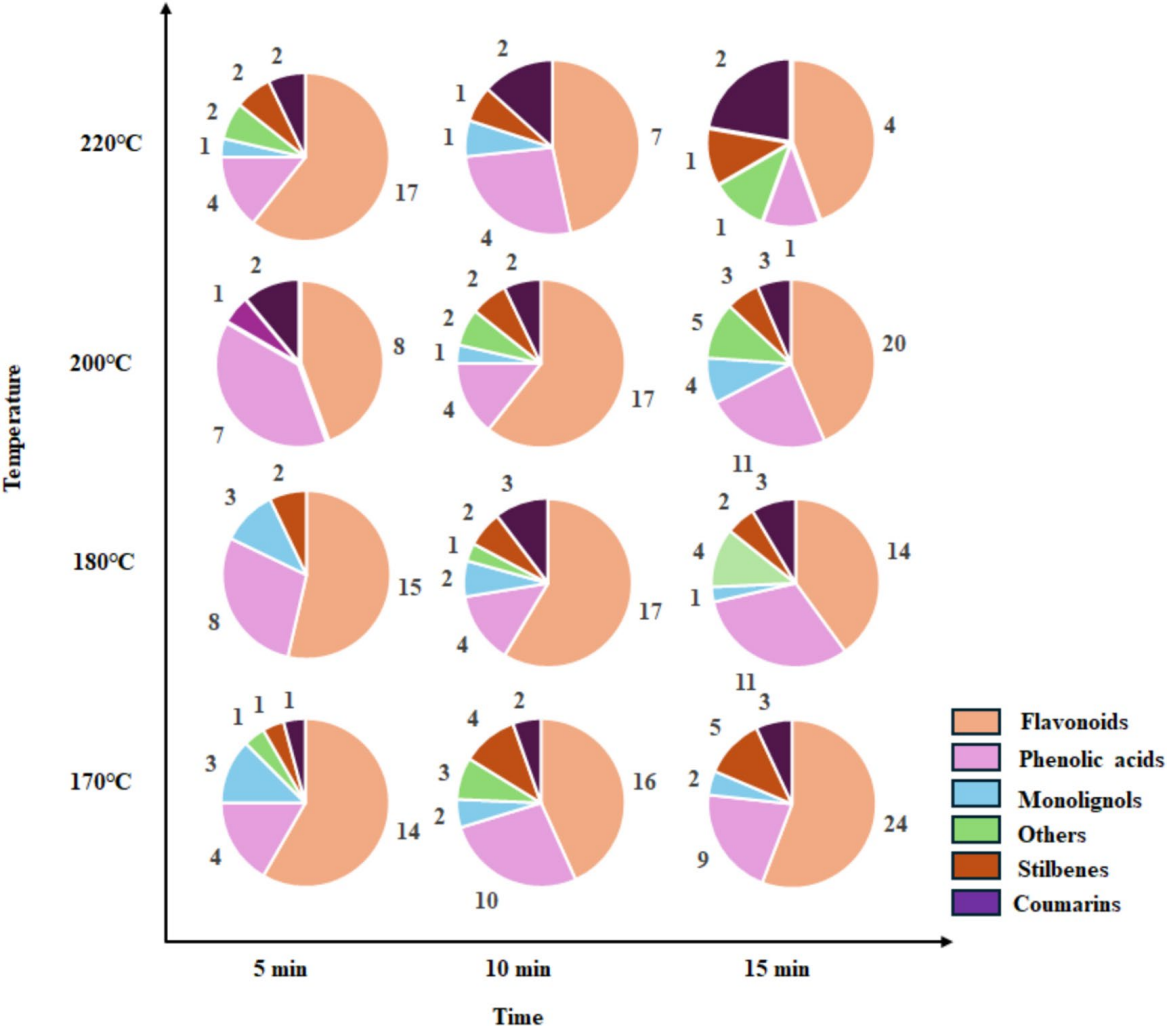
Variations in phenylpropanoid content in kānuka subcritical water extract at different temperatures and extraction times

The compounds identified in kānuka extracts were categorised based on their chemical configuration. The diversity of compounds identified at different SWE conditions is presented in Fig. 5. The major bioactives in the kānuka extracts are flavonoids and phenolic acids, as noted by Essien et al. [9]. Minor groups include stilbenes, monolignols, and coumarins. The control sample is rich in flavonoids [19] and phenolic acids [9], followed by other compounds, such as precursor of lignin (coniferyl alcohol) and aldehyde (Hydroxybenzaldehyde). However, the compounds belonging to stilbenes [2], coumarins [2], and monolignols [1] are relatively low. The diversity of compounds identified at different SWE conditions is presented in Fig. 6. Results suggest that maximum flavonoid recovery can be obtained at 170 °C and 200 °C. Phenolic acids are the second most abundant in kānuka extracts. However, the maximum recovery varies based on temperature and time, where lower temperature (170 °C) requires a longer time (15 min) and higher temperature (200 °C) requires a shorter extraction time (5 min). Overall, the maximum number of phenylpropanoids are recovered at 170 °C, 15 min (43 compounds) and 200 °C, 15 min (46 compounds), correlating with the results obtained in TPC and TFC assays. Stilbenes and coumarins occurred consistently throughout the extractions, while monolignols gradually decreased at a higher temperature of 220 °C.

The bioactive compounds identified at 170 °C (5, 10, 15 min) using positive and negative ionisation are presented in this report (Table 2). Compounds identified at other temperatures (180 °C, 200 °C, and 220 °C) and fragmentation patterns are presented in Supplementary Table 1 and Supplementary Table 2, respectively. It is important to note that in this study, compound identification was conducted using a personal compound library database. The database predominantly focuses on flavonoids and phenolic acids, while other compounds were less extensively represented. Hence, some variation can be expected when comparing the outcomes of the quantitative analysis with the qualitative results.

**Table 2:** Bioactive compounds of kānuka subcritical water extract obtained at 170 °C and 5 min, 10 min, and 15 min intervals identified using LC–MS/MS-QTOF with positive and negative ionisation

Compounds	Molecular formula	Theoretical mass (g/moL)	Observed mass (g/moL)	Retention time (min)	Isotope (m/z)	Error difference (ppm)	ID. Score (%)	Species
*Flavonoids*								
2′,6′-Dihydroxy-4′- methoxydihydrochalcone	C_16_ H_16_ O_4_	272.1049	272.1051	7.362	273.1127	0.81	64.97	(M+H)+
Poncirin	C_28_ H_34_ O_14_	594.1949	594.1967	8.541	595.2047	3.12	63.72	(M+H)+
Apigenin 7-*O*-[beta-d-apiosyl-(1- > 2)-beta-d-glucoside]	C_26_ H_28_ O_14_	564.1479	564.1472	6.858	565.1552	−1.17	68.52	(M+H)+
Baicalein 5,6,7-trimethyl ether	C_18_ H_16_ O_5_	312.0998	312.0998	11.968	313.1073	−0.07	67.83	(M+H)+
Baicalin	C_21_ H_18_ O_11_	446.0849	446.0838	6.551	447.0927	1.16	81.01	(M+H)+
4′,5,6,7-Tetramethoxyflavone	C_19_ H_18_ O_6_	342.1103	342.1105	14.396	343.1187	0.51	67.35	(M+H)+
Cyanidin 3,5-*O*-diglucoside	C_27_ H_31_ O_16_	611.1612	611.1625	14.205	611.1624	2.12	59.56	(M+H)+
Scutellarein	C_15_ H_10_ O_6_	286.0477	286.0478	14.082	286.0475	0.21	76.91	(M−H)−
( +)-Gallocatechin	C_15_ H_14_ O_7_	306.074	306.0743	4.071	305.0673	1.25	89.02	(M−H)−
Fustin	C_15_ H_12_ O_6_	288.0634	288.0634	14.884	287.0572	−0.06	74.66	(M−H)−
Tectochrysin	C_16_ H_12_ O_4_	268.0736	268.0734	4.025	267.066	−0.7	82.22	(M−H)−
Morin	C_15_ H_10_ O_7_	302.0427	302.0412	11.794	302.038	−4.82	92.02	(M−H)−
Gardenin B	C_19_ H_18_ O_7_	358.1053	358.1066	1.696	357.1007	3.84	68.81	(M−H)−
Garbanzol	C_15_ H_12_ O_5_	272.0685	272.0685	7.964	271.0613	0.18	85.47	(M−H)−
Quercetin 3 -*O*-(6″-malonyl glucoside)	C_24_ H_22_ O_15_	550.0959	550.0972	1.734	549.0876	2.47	64.73	(M−H)−
( +)-Catechin	C_15_ H_14_ O_6_	290.079	290.0782	17.101	291.0858	−2.88	61.71	(M+H)+
5-*O*-Caffeoylshikimic acid	C_16_ H_16_ O_8_	336.0845	336.0832	3.948	337.0904	−3.83	79.4	(M+H)+
Gossypetin	C_15_ H_10_ O_8_	318.0376	318.0364	9.025	317.0292	−3.53	95.56	(M−H)−
*Flavonoids*								
Chrysoeriol 7-*O*-glucoside	C_22_ H_22_ O_11_	462.1162	462.1167	1.985	461.1077	1.1	60.83	(M−H)−
Pinobanksin 3-*O*-acetate	C_17_ H_14_ O_6_	314.079	314.079	17.519	313.0714	−0.1	76.09	(M−H)−
Vitexin	C_21_ H_20_ O_10_	432.1056	432.1035	11.211	431.0968	−4.94	60.37	(M−H)−
Hispidulin	C_16_ H1_2_ O_6_	300.0634	300.0626	16.774	300.0585	−2.74	80.87	(M−H)−
7-Hydroxyflavone	C_15_ H_10_ O_3_	238.063	238.0624	7.921	239.069	−2.62	73.98	(M+H)+
Tricin	C_17_ H_14_ O_7_	330.074	330.0732	15.614	331.0806	−2.29	74.71	(M+H)+
Silandrin	C_25_ H_22_ O_9_	466.1264	466.1268	14.874	465.1199	1	75.7	(M−H)−
*Phenolic acids*								
Ellagic acid	C_14_ H_6_ O_8_	302.0063	302.0053	6.146	300.9981	−3.14	96.04	(M−H)−
Gallic acid	C_7_ H_6_ O_5_	170.0215	170.0211	8.184	169.0138	−2.63	86.03	(M−H)−
Chlorogenic acid	C_16_ H_18_ O_9_	354.0951	354.0953	1.696	353.0878	0.72	78.62	(M−H)−
Caffeic acid	C_9_ H_8_ O_4_	180.0423	180.0421	4.025	179.0345	−0.98	88.78	(M−H)−
Sinapic acid	C_11_ H_12_ O_5_	224.0685	224.0675	19.447	223.0603	−4.39	82.36	(M−H)−
3-p-Coumaroylquinic acid	C_16_ H_18_ O_8_	338.1002	338.0985	3.766	337.0912	−4.81	75.5	(M−H)−
Quinic acid	C_7_ H_12_ O_6_	192.0634	192.0641	3.958	191.057	3.63	78.1	(M−H)−
4-Sinapoylquinic acid	C_18_ H_22_ O_10_	398.1213	398.121	5.865	397.1143	−0.81	65.04	(M−H)−
3-Feruloylquinic acid	C_17_ H_20_ O_9_	368.1107	368.1094	3.092	367.1019	−3.5	73.5	(M−H)−
2-Hydroxybenzoic acid	C_7_ H_6_ O_3_	138.0317	138.031	3.713	137.0237	−4.73	84.52	(M−H)−
Isochlorogenic acid b	C_25_ H_24_ O_12_	516.1268	516.1235	10.342	517.1314	−6.37	56.24	(M+H)+
*Coumarins*								
Daphnetin	C_9_ H_6_ O_4_	178.0266	178.026	15.152	179.033	−3.31	76	(M+H)+
Scopoletin	C_10_ H_8_ O_4_	192.0423	192.0416	15.128	194.0509	−3.66	79.63	(M+H)+
Isopimpinellin	C_13_ H_10_ O_5_	246.0528	246.0525	16.268	247.0598	−1.38	84.94	(M+H)+
*Others*								
4-Coumarate	C_9_ H_8_ O_3_	164.0473	164.0467	2.831	164.0429	−4.2	84.99	(M−H)−
Dihydroconiferyl alcohol	C_10_ H_14_ O_3_	182.0943	182.0936	10.659	181.0864	−3.62	97.58	(M−H)−
6-Gingerol	C_17_ H_26_ O_4_	294.1831	294.1828	25.502	293.1757	−0.89	85.36	(M−H)−
4-Hydroxybenzaldehyde	C_7_ H_6_ O_2_	122.0368	122.0371	5.715	123.0447	2.48	59.66	(M+H)+
Coniferyl aldehyde	C_10_ H_10_ O_3_	178.063	178.0627	23.898	177.055	−1.76	71.4	(M−H)−
Mangiferin	C_19_ H_18_ O_11_	422.0849	422.0852	8.925	421.0787	0.58	69.25	(M−H)−
*Monolignols*								
Todolactol A	C_20_ H_24_ O_7_	376.1522	376.1518	6.021	375.1448	−1.11	69.71	(M−H)−
Matairesinol	C_20_ H_22_ O_6_	358.1416	358.1406	7.812	359.1461	−2.99	71.47	(M+H)+
Coniferyl alcohol	C_10_ H_12_ O_3_	180.0786	180.0783	6.719	179.0703	−2.02	58.31	(M−H)−
*Stilbenes*								
Pinosylvin	C_14_ H_12_ O_2_	212.0837	212.0836	10.787	211.0766	−0.52	93.25	(M−H)−
Piceatannol	C_14_ H_12_ O_4_	244.0736	244.0725	2.859	245.0793	−4.36	68.54	(M+H)+
Astringin	C_20_ H_22_ O_9_	406.1264	406.1273	9.025	405.1191	2.25	71.4	(M−H)−
Resveratrol	C_14_ H_12_ O_3_	228.0786	228.0779	19.565	227.0707	−3.07	96.63	(M−H)−

Frequently detected compounds in triplicate (*n* = 3) are listed

This analysis revealed the presence of common tea tree plant phenylpropanoids, including ellagic acid (m/z: 300.9999 (M−H)−), chlorogenic acid (m/z: 355.1017 (M−H)−), ( +)-catechin (m/z: 289.0735 (M−H)−), and epigallocatechin gallate (m/z: 457.0774 (M−H)−) in kānuka water extract (control). These compounds are most commonly present in medicinal and edible plants, demonstrating antioxidant, antimicrobial, anti-diuretic, and anti-inflammatory properties [64–66]. Additionally, SWE facilitated the identification of previously unreported compounds, including astringin (m/z: 405.1224 (M−H)−), dihydroconiferyl alcohol (m/z: 181.0879 (M−H)−), 3-methylxanthine (m/z: 165.0421 (M−H)−), scutellarein (m/z: 286.0454 (M−H)−), gossyptein (m/z: 319.0443 (M+H)+), and piceatannol (m/z: 246.0808 (M+H)+). These findings demonstrate the efficiency of SWE in recovering diverse bioactive molecules with varying polarity.

Furthermore, compounds identified in control samples, including ellagic acid, baicalin (m/z: 445.0762 (M−H)−), poncirin (m/z: 595.2033 (M+H)+), isopimpinellin (m/z: 247.0604 (M+H)+), gossypetin, were found at different extraction temperatures demonstrating their stability at elevated subcritical water temperature. However, depending on their physicochemical properties, the ionic strength of the compounds varies at different temperatures (Supplementary online resource 3). For instance, the relative abundance (intensity of ion) for compounds in control, including gossypetin, ellagic acid, baicalin, and isopimpinellin, increased at 170 °C from 664.84, 11,961.03, 2084.03, 264.94 to 5920.36, 358,812.88, 2875.79, and 648.18, respectively. In contrast, the relative abundance of the poncirin molecule decreased at 170 °C to 460.12 compared to the control (1671.08).

Additionally, compounds observed in control samples, including procyanidin B2 (m/z: 577.1358 (M−H)−) and epigallocatechin gallate, were not found at higher temperatures (170–220 °C) due to their heat instability. Wahydiono et al. [67] observed a gradual decrease in the recovery of procyanidin B2 from apple peel using SWE above 150 °C, suggesting that the minimum threshold temperature required for the decomposition is 150 °C. Similarly, Ko et al. [68] observed a decrease in the recovery of epigallocatechin gallate from green tea leaves using SWE at 110 °C. Procyanidin B2 is a dimeric proanthocyanidin, consisting of two flavan-3-ol units linked through either C4 → C8 or C4 → C6 bond, while epigallocatechin is a monomeric flavan-3-ol [69]. According to Dallas et al. [70], thermal energy initiates the degradation of proanthocyanidins B2, generating monomeric flavan-3-ol, such as catechin. Further degradation of flavan-3-ol may result in smaller compounds, like phenolic acids [69, 70]. Hence, the increased phenolic acids and flavonoids recovery at increasing temperatures might result from the degradation of polymeric compounds, including procyanidins B2 and epigallocatechin.

#### Flavonoids

Flavonoids are one of the most predominant groups of polyphenolic metabolites in plants. Their core structure consists of two benzene rings connected by three-carbon pyran (C_6_–C_3_–C_6_). Based on the type of functional group (hydroxyl, methoxy, glycoside, and ketone) attached to the core structure, they can be classified as flavonols, flavones, flavanones, flavanols, anthocyanidins, and isoflavones [71].

The optimum extraction temperature of flavonoids is determined by the structural relationship between the solute and solvent (water) at higher temperatures and pressure [58, 72]. For instance, compounds with more hydroxyl groups (myricetin and quercetin) can be extracted at temperatures between 160 and 170 °C, whereas compounds with fewer hydroxyl groups (kaempferol) require 200 °C [73]. Likewise, in kānuka, the compounds with different functional groups, including double bond (7-hydroxyflavone), 6-OH groups (( +)-gallocatechin), sugar (cyanidin 3,5-*O*-diglucoside and chrysoeriol-7-*O*-glucoside), hydroxyl and methylated groups (poncirin and 2ʹ,6ʹ-Dihydroxy-4ʹ-methoxy dihydrochalcone) were identified between 170 and 200 °C. These findings are consistent with the studies of Ko et al. [58] and Cheigh et al. [73].

Identifying bioactive molecules is challenging due to the complex mixture present in plant extracts. However, combining the soft ionisation (electrospray ionisation) technique with collision-induced dissociation (CID) and tandem mass spectrometry (MS/MS) offers a comprehensive structural analysis of the compounds. This approach generates molecular (product) ions and their fragments, providing valuable insights into the structure of the compounds. Fragmentation ions (FI) are produced from molecular ions through CID, where cleavage occurs at different positions within the molecules (C–C and C–H). Several factors influence the formation of fragmentation patterns, including ionisation mode ((M+H)+ or (M−H)−), collision energy, and degree of hydroxylation. In addition, compounds with more hydroxyl groups form distinct fragments rather than an unsubstituted B ring, requiring higher cleavage energy [74].

**Fig. 7:**
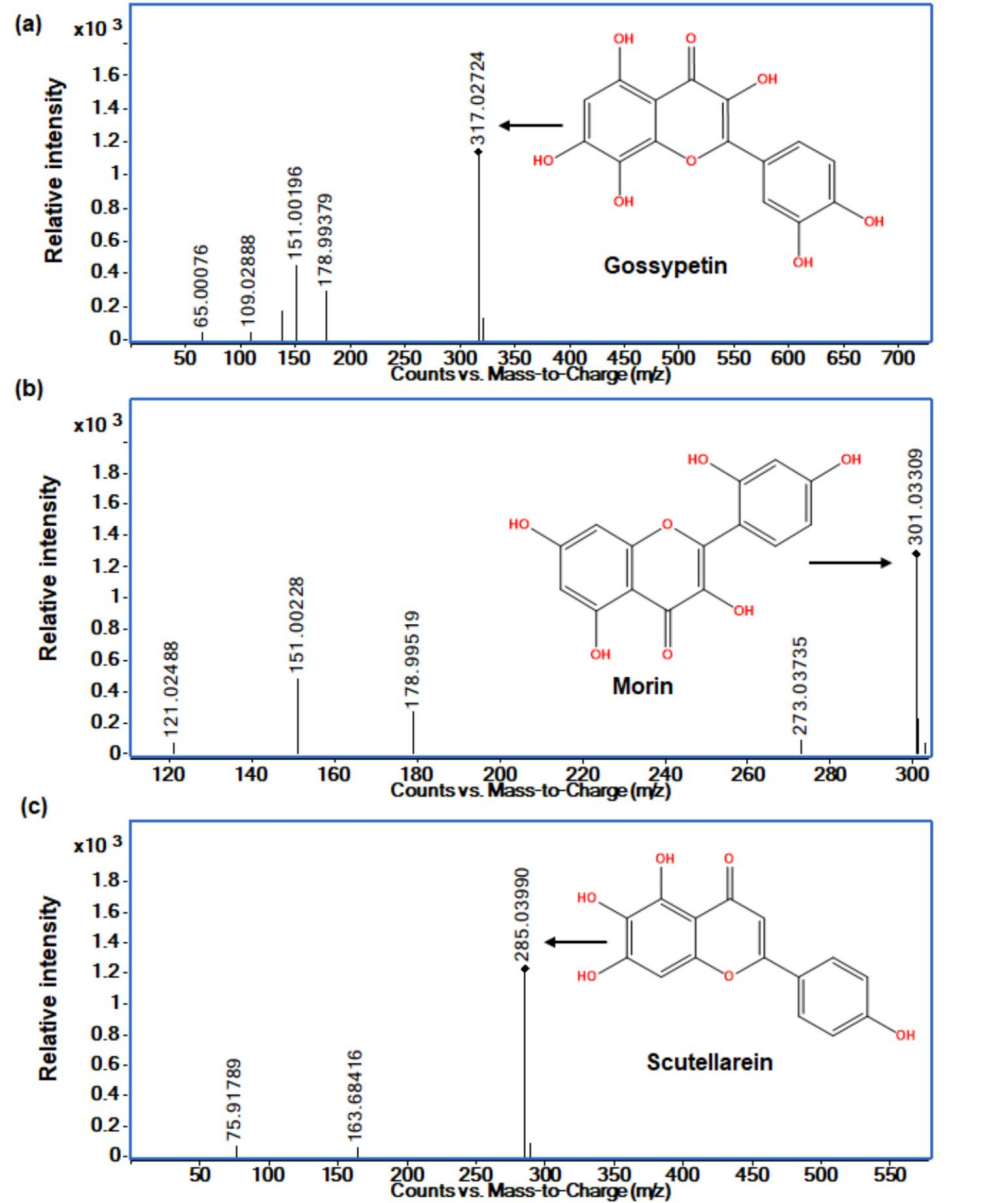
Fragmentation pattern (MS/MS) of the flavonoids identified in kānuka subcritical water extracts at 170 °C. **a** Gossypetin (M−H)−, **b** morin (M−H)−, and **c** scutellarein (M−H)−. Black diamond suit -product ion

**Fig. 8:**
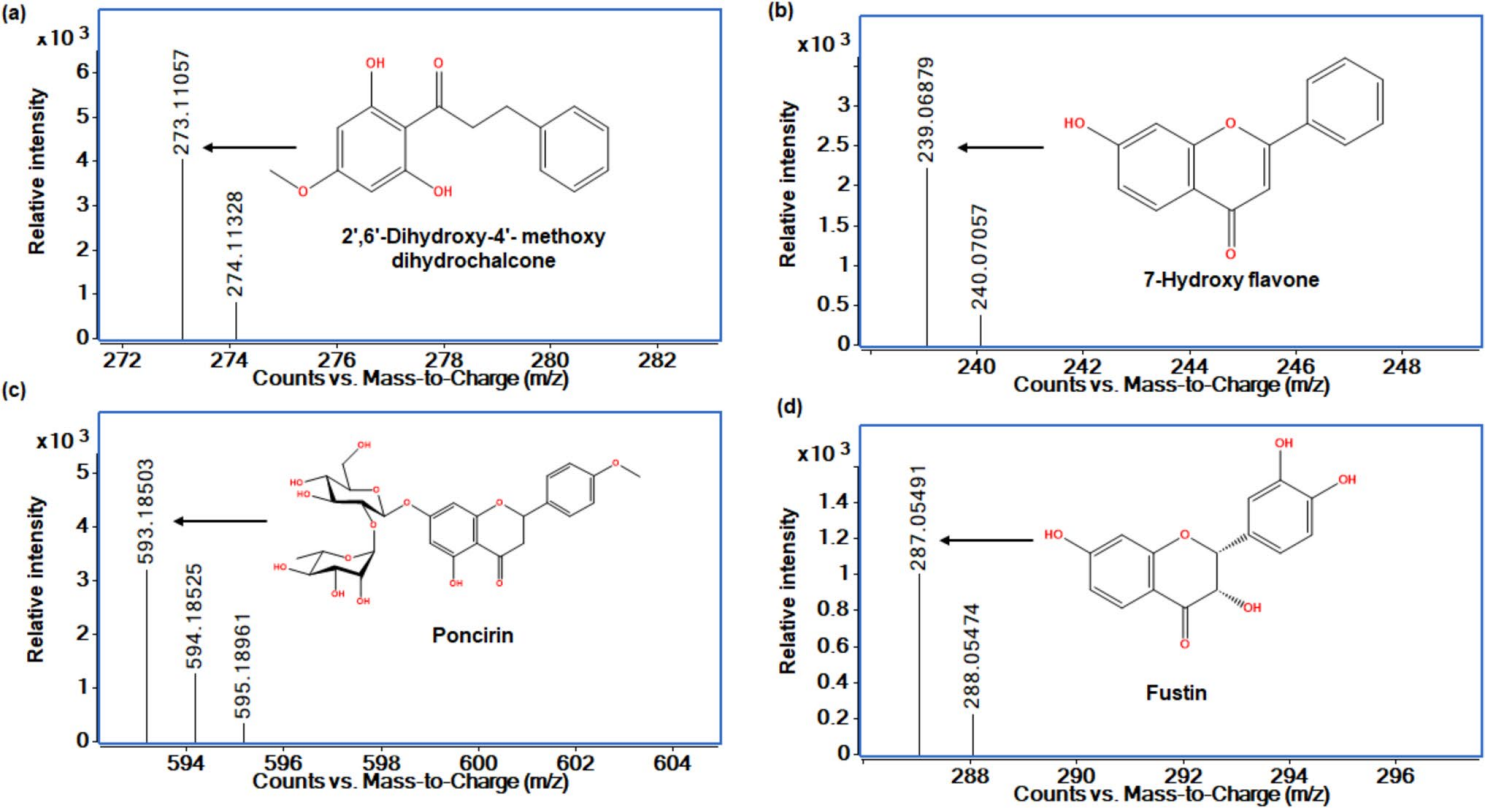
Mass spectrum of flavonoids identified in kānuka subcritical water extracts at 170 °C. **a** 2′,6′-Dihydroxy-4′- methoxy dihydrochalcone (M+H)+, **b** 7-hydroxy flavone (M+H)+, **c** poncirin (M−H)+, and **d** fustin (M−H)−

In kānuka extracts, flavonoids containing more hydroxyl groups, including gossypetin (C-3, 3′, 4′, 5, 7, and 8) (m/z: 317.0292 (M−H)−; FI m/z: 151.00196) and morin (C-2′, 4′, 5) (m/z: 302.0370 (M−H)−; FI m/z: 151.00228) were fragmented (Fig. 7), while poncirin and 7-hydroxyflavone were not fragmented (Fig. 8) at given collision energy, 15 eV. Similarly, the compound scutellarein (m/z: 286.0475, (M−H)−) was fragmented when the (M−H)− precursor ion was used compared to (M+H)+. These outcomes demonstrate that the influence of collision energy applied was insufficient to produce the fragmentation of flavonoids with high molecular mass regardless of functional group, as reviewed by Cuyckens and Claeys [74].

*Potential health benefits of kānuka flavonoids:* Identifying biologically potent flavonoids in kānuka extracts highlights their possible contribution to drug discovery. For instance, 2′,6′-Dihydroxy-4′- methoxy dihydrochalcone (DHMDC) and fustin (flavanonol) have been studied for their inhibitory effects on acetylcholinesterase (AChE), an enzyme associated with AD. According to Gonçalves et al. [75], oral administration of DHMDC (30 mg/kg) reduced more than 50% of the development of cognitive deficits in male Swiss albino AD mice (in vivo) induced with streptozotocin. The effect was comparable to the control group treated with anti-AD drug rivastigmine (0.6 mg/mL). Furthermore, their molecular interaction study between DHMDC and AchE revealed that DHMDC inhibits the protein via interacting with active site residues, including hydrogen bond, π-, CH…π and Van der Waals interactions at Phenylalanine295, Tyrosine337, Trptophan286, and Tyrosine83, respectively. Fustin exhibits a protective mechanism via the muscarinic M1 receptor and ERK signalling pathway, which are crucial for memory function. It has been demonstrated to restore memory function in C57BL/6 mice impaired with amyloid-beta peptide (Aβ (1–42) and Aβ (42–1)) at the concentration of 400 pmol/5 L compared to EGb761 (50 mg/mL) [76]. Similarly, in kānuka, Majid & Silva [26] observed 62.9% inhibition of β-secretase enzyme associated with AD using ultrasound-assisted extract (IC_50_ 14.25 µg/mL). Identifying these bioactive metabolites provides compelling evidence that kānuka flavonoids can be an effective drug metabolite for treating AD. These findings underscore the value of kānuka-derived flavonoids as promising candidates in developing neuroprotective drugs that target multiple pathways associated with cognitive decline.

Poncirin, a disaccharide derivative of flavanone glycoside known for its bitter taste, has demonstrated several health benefits, such as anti-inflammatory effects. Kim et al. [77] observed the anti-inflammatory effect of poncirin is dose-dependent, where increasing concentrations (100 µM) required to reduce the expression of proinflammatory factors, such as tumour necrosis factor-α (< 4 ng/mL) and interleukin-6 (< 1 ng/mL), in RAW 647.7 macrophage cell line, induced with lipopolysaccharide (1 µg/mL), compared to untreated cells. Similarly, the in vitro and in vivo study of Yoon et al. [78] demonstrated that poncirin (3 mg/kg/day) significantly enhanced the structural properties of trabecular bone and mineral density (BV/TV ratio with a + 31.4% increase, a measure of trabecular bone volume to the total volume of space it occupies) and reduced bone resorption in glucocorticoid-induced (2.1 mg/kg/day) male ICR mice compared to the positive control group (Genistein 10 mg/kg/day, BV/TV: + 18.2%). The identification of poncirin in kānuka aligns with traditional practices, where a decoction of bark (boiled in water) has been prescribed (at a dosage of a cupful twice a day) for fracture healing and pain relief [79]. This outcome suggests that bioactive compounds in kānuka can offer a potential alternative in therapeutic applications targeting inflammation-related diseases and improving skeletal function.

7-Hydroxyflavone, a hydroxy flavonoid, is known for its potential health-promoting effects, including antioxidant, antiproliferative, and antibacterial effects. 7-hydroxyflavone purified from *Avicennia officinalis* demonstrated a dose-dependent (in vitro) anti-tumour effect on human cervical (HeLa) (IC_50_ 22.56 ± 0.21 µg/mL) and breast cancer cell lines (MDA-MB231) (IC_50_ 3.86 ± 0.35 µg/mL). This antiproliferative activity is attributed to 7-hydroxyflavone's higher affinity for negatively charged amino acid residues, including Threonine55, Alanine59, Phenylalanine63, and Glutamine58 in the Bcl-2 (B-cell lymphoma 2- groove site) via hydrophobic interaction to induce the conformational changes to form a stable ligand-target complex [80, 81]. Similarly, studies have demonstrated that kānuka exhibits dose-dependent antiproliferative activity, which is selective to the type of cell line. For example, the extract obtained at 210 °C effectively inhibited the proliferation of murine fibroblast (IC_50_ 216.8 µg/mL), human rhabdomyosarcoma (IC_50_ 389 µg/mL) and human cervix carcinoma (IC_50_ 317.7 µg/mL) cell lines. Likewise, the kānuka extract (100 µg/mL) rich in flavonoid glycoside exerted 90% inhibition on mouse lymphoblastic parental P388 tumour cells [25, 27]. This selectivity is advantageous in drug development as it allows the metabolite to target the cancer cells, thereby reducing damage to non-cancerous cell lines.

*Bitterness and Beyond*: The taste of green tea encompasses bitterness, sweetness, astringency, and a brothy taste, which are attributed to its unique chemical constituents. Notably, the infusion of young shoots tastes more bitter than dried leaves [82, 83]. Similarly, kānuka leaf infusions prepared with fresh leaves exhibit a stronger bitterness than those made with dried leaves [14]. This observation aligns with the bioactive profile identified in kānuka. Catechin and gallocatechin, known contributors to bitterness in tea [84], were present in the control sample. However, their relative intensity varied depending on the extraction temperatures. For instance, the intensity of catechin was higher at room temperature (3329.32), decreased at 170 °C (1642.66) and increased again at 200 °C (2411.41). This variation demonstrates the stability and solubility of catechin at different temperatures. At 170 °C, catechin might have started to degrade, resulting in reduced intensity. However, the increased solubility of solvent at higher temperatures might have enhanced the solubility of catechin, resulting in increased intensity at 200 °C.

Additionally, the presence of naringin (flavone glycoside) at higher temperatures (180 °C, 200 °C, and 220 °C) might contribute to the bitterness of kānuka extract. Some bitter compounds, like denatonium, possess therapeutic potential, activating bitter taste receptors (TAS2R) in taste buds and non-sensory tissues acting as bronchodilators. According to Ni et al. [85], naringin (0.1 mm to 1.0 mM) can promote the proliferation of human bronchial epithelial cells (16HBE14o) by influencing the expression of cyclin B and E proteins, which are involved in the progression of the cell cycle. It is in contrast to denatonium, which inhibits proliferation. Bronchial epithelial cell proliferation helps repair and maintain the bronchial lining during bronchitis. This outcome supports the traditional practice of consuming kānuka leaves decoction to treat bronchitis [14], highlighting the therapeutic synergy between kānuka’s traditional uses and modern pharmacological discoveries.

*Promiscuous compounds:* Bioactive compounds from plants have received increasing attention in drug development due to their ability to interact with many proteins that cause diseases [86]. However, this non-selective mode of interaction may not always produce beneficial effects. There is an increasing chance for the bioactive functional group to interact with untargeted molecules/proteins, causing damage that leads to detrimental impacts. This multipotential ability needs to be analysed to avoid the selection of compounds with promiscuous behaviour for further optimisation, which often produces fruitless results [87].

One advantage of qualitative screening of plant metabolites is that it helps avoid selecting compounds with promiscuous behaviour for further analysis or optimisation. Some flavonoids in kānuka extracts, including quercetin derivatives and compounds with catechol groups, have been noted to exhibit promiscuous behaviour, including quercetin-3’-*O*-glucuronide, (+)- catechin, (+) gallocatechin, epigallocatechin gallate, quercetin −3-*O*-(6″-malonyl glucoside), quercetin-3-*O*-glucosyl-xyloside, and resveratrol. According to Baell [88], molecules containing a common substructural moiety, catechol, interfere with the assays differently, leading to false positive results that do not reflect true drug potential. Hence, careful consideration is needed when selecting bioactives for specific biological applications. Despite these challenges, many of these compounds have demonstrated significant health benefits. For example, derivatives of quercetin and epigallocatechin have shown potential antioxidant, anti-inflammatory, anti-tumour, and cardiovascular health properties [89]. This targeted approach enhances the reliability of kānuka extracts for specific biological applications and ensures their suitability for developing effective therapeutic agents.

As a result, tentative identification of flavonoids in kānuka extracts has evidenced the presence of biologically important bioactives with possible applications in different industrial sectors, including pharmaceuticals, food, and cosmetic.

#### Phenolic Acids

The phenolic acids constitute an aromatic group of phenolic compounds with a benzene ring linked to one or more hydroxy or methyl groups, and they are divided into hydroxycinnamic and hydroxybenzoic acids [63]. In kānuka, fewer phenolic acids have been detected compared to flavonoids. These findings differ from the TPC quantification outcome, where TPC is relatively higher. Fabian et al. [52] observed a similar trend, where HPLC analysis and TPC results in rice bran were not statistically significant. Additionally, they investigated the thermal stability of common phenolic acids, including gallic acid, ferulic acid, caffeic acid, and *p-*coumaric acid. They reported that these compounds likely underwent decomposition via decarboxylation and dehydration at a minimum of 175 °C. Furthermore, ferulic acid was identified as the least resistant to elevated temperatures.

In kānuka, at 170 °C, phenolic acids, including derivatives of benzoic acids and quinic acids, are predominant, followed by gallic acid, caffeic acid (m/z: 179.0345, (M−H)−), and chlorogenic acid (m/z 353.0878, (M−H)−). The relative ion intensity varies depending on the compound. For example, gallic acid exhibits a decreasing trend in ion intensity (from 61,692.37 to 42,968.48), whereas the ion strength for ellagic acid increases significantly (from 11,961.03 to 358,812.88) at higher temperatures. It indicates that the degradation of gallic acid has initiated and led to an increased concentration of ellagic acid, as Fabian et al. [52] noted. Similarly, ferulic acids are absent in the extract at temperatures above 170 °C. Instead, a decarboxylation product, ferulate, is found in the extracts at 180 °C, 200 °C, and 220 °C.

**Fig. 9:**
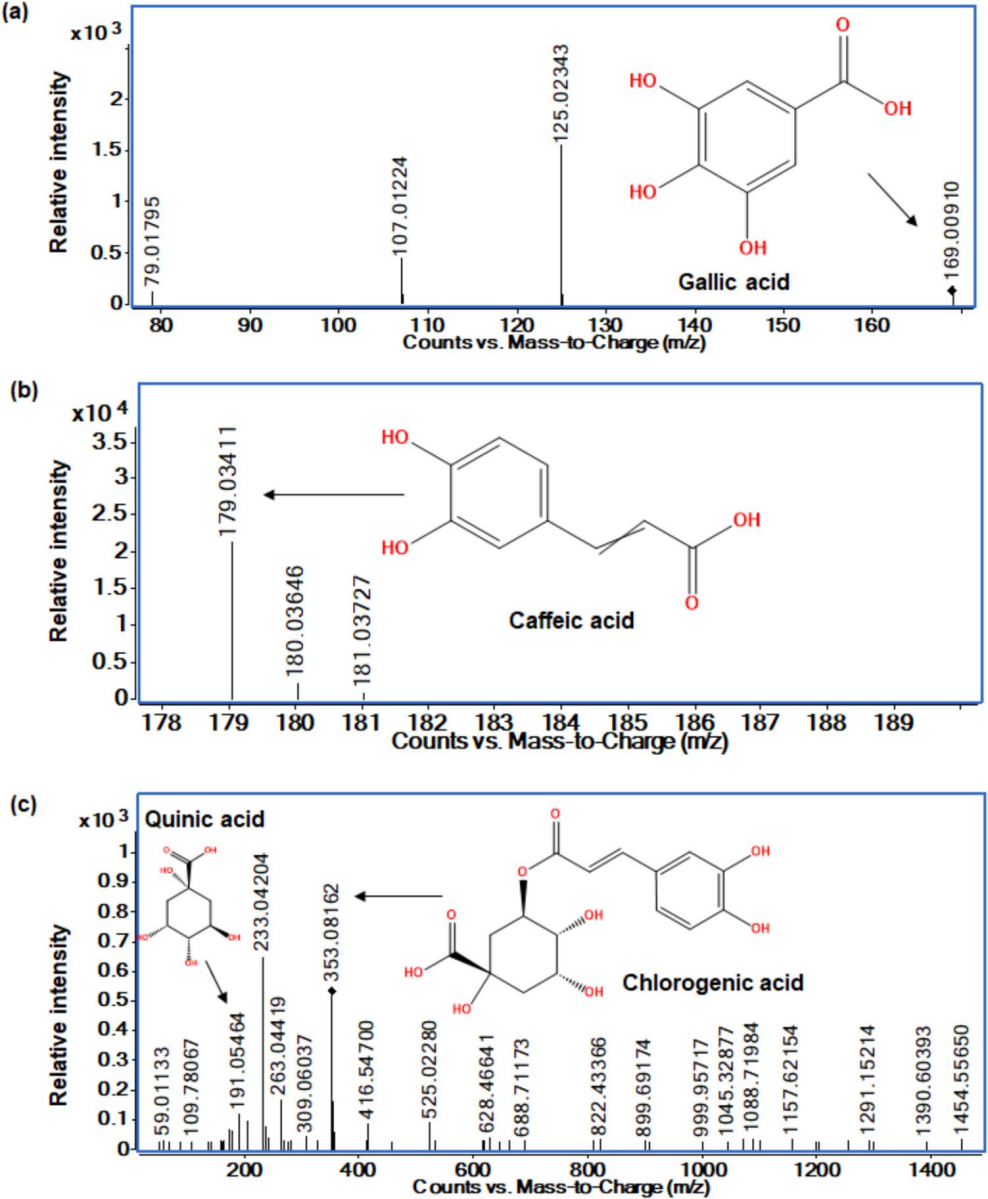
Kānuka phenolic acids obtained at 170 °C using negative ionisation mode at 15 eV collision energy **a** Gallic acid (MS/MS), **b** caffeic acid (MS), and **c** chlorogenic acid (MS/MS)

The applied collision energy of 15 eV induced fragmentation of kānuka phenolic acids (Fig. 9) in negative ionisation mode. Fragmentation of gallic acid (m/z 169.00910; FI m/z: 125.02343) has occurred with the removal of CO_2_, producing a fragment at m/z 125.02343, which further fragmented with the loss of H_2_O, resulting in m/z 107.01124, as noted by [90, 91]). The fragment at m/z 79.0179 resulted from the release of CO from m/z 107.01124.

The esterification of caffeic and quinic acid forms chlorogenic acid [92]. The deprotonated molecular ion of chlorogenic acid was observed at m/z 353.08162 (FI m/z: 191.05464). Initial fragmentation commenced at the caffeic acid moiety, releasing CO_2_ (m/z 309.06037), CO, and H_2_O (m/z 263.04419). The release of quinic acid at m/z 191.05464 indicates that the fragmentation occurred at the site where the caffeic acid is bound to the (5th OH group) quinic acid, as Pearson et al. [93] noted.

Phenolic acids are renowned for their health benefits. Ellagic and chlorogenic acids, in particular, are important polyphenolic compounds that have gained substantial research interest in elucidating the molecular mechanism due to their various biological activities, especially in renal function. Diabetes mellitus (DM) is a major risk factor causing kidney disease. Several studies have reported that high serum glucose levels (hyperglycaemia) in blood potentially induce NF-kB transcriptional factors, leading to renal fibrosis in patients with DM [64, 65].

Ahad et al. [64] demonstrated that ellagic acid has significantly improved the functional properties of the kidney, including glomerular filtration rate. Their findings revealed that ellagic acid effectively increased the creatinine clearance rate (0.8 mL/min) and decreased the serum creatinine (0.5–1 mg/dL), proteinuria (100 mg/24 h), and blood urea nitrogen (10–20 mg/dL) level in diabetic adult male albino rats induced with streptozotocin (35 mg/kg BW) compared to the diabetic control group (0.2 mL/min, 2.5 mg/mL, 600 mg/dL, and 70 mg/dL, respectively).

Similarly, studies on cherry stem extract (decoction), which is rich in derivatives of chlorogenic acid (quinic acid, caffeic acid), quercetin, and kaempferol, have demonstrated potential diuretic properties by increasing urine excretion in adult male Charles River Wistar rats [66]. Notably, the major flavonoids and phenolic acids identified in the cherry stem decoction are observed in the bioactive profile of kānuka extracts. This similarity offers key chances in investing the possible diuretic application of kānuka metabolites.

Consequently, the identified phenolic acids in kānuka extract and studies on its functional properties related to kidney disease [64–66] underscores the validation of traditional practices involving kānuka decoction in treating kidney and bladder ailments [14, 15].

#### Stilbenes and Monolignols

Stilbenes are phenylpropanoid molecules characterised by a 1,2-diphenylethylene core structure (C_6_–C_2_–C_6_), found only in certain plant families, such as Myrtaceae. Four different stilbenes, including pinosylvin, piceatannol, astringin, and resveratrol, are found in kānuka extracts ranging from 170 to 200 °C. However, at 220 °C, the number of stilbenes has reduced significantly. Most prevalent stilbene is piceatannol. It indicates that the optimum conditions for extracting stilbenes are 170–200 °C. These findings partially align with Gabaston et al. [94], who noted that the optimum SWE temperature for the maximum recovery of piceatannol and resveratrol is 160 °C (5–30 min) from grapevine by-products. Like other phenolic compounds, stilbenes are well-known for their antioxidant, anti-inflammatory, anti-acne, anti-ageing, and anti-cancer properties.

Monolignols serve as fundamental constituents in the synthesis of lignins and lignans. The primary monomers involved in lignan synthesis are *p*-coumaryl alcohol, sinapyl alcohol, and coniferyl alcohol [63]. They are the derivatives of phenylpropanoid metabolism found in relatively lower proportions than other metabolites. Lignans, todolactol A, and matairesinols have been identified in kānuka extract between 170 and 200 °C. These compounds are well-documented for various health benefits, including cancer prevention and cardiovascular health [95]. The presence of other monolignols, including coniferyl alcohol and 4-coumaryl alcohol, indicates the formation of lignans and their stability at higher temperatures. Meanwhile, the absence of these monolignols indicates that the formation of lignans has been gradually reduced at 220 °C. These findings are consistent with the study of Özkaynak Kanmaz and Ova [96], who reported that the maximum recovery of lignan (secoisolariciresinol diglucoside) obtained at 180 °C using solvent-accelerated SWE from flaxseed meal stick. The identification of lignan in kānuka supports their incorporation into functional foods or nutraceuticals. Furthermore, the temperature-dependent behaviour of monolignols suggest a pathway for optimising the extraction of these health-promoting lignans for clinical applications.

## Conclusion

Kānuka, a traditional medicinal plant of New Zealand, holds promise for its in vitro biological properties. The qualitative analysis of kānuka subcritical water extract revealed diverse phenylpropanoids, including flavonoids, phenolic acids, stilbenes, and monolignols, highlighting its potential for translational research. Subcritical water extraction demonstrated the potential for a sustainable and environmentally friendly method for extracting bioactive compounds, offering pathways for green chemistry and resource recovery.

The identified metabolites, particularly flavonoids and phenolic acids, demonstrate promising therapeutic potential and offer researchers new insights for various applications. For example, these metabolites can support the development of drugs targeting chronic diseases, infection, and inflammation conditions. The extract’s antioxidant properties can serve as a potential candidate for nutraceutical supplements and as an active ingredient in cosmetic formulations to promote anti-ageing effects and address skin conditions like acne. Additionally, these compounds hold promise as functional ingredients in the food and beverage industry, enhancing flavour and health-promoting properties.

Notably, this study establishes a strong association between in vitro biological studies based on mātauranga Māori and the biological properties of the compounds identified. It highlights a potential bridge between the Māori traditional practice and scientific findings, highlighting the cultural and scientific significance of kānuka. Further research on exploring the synergistic effect of the extract with other plant metabolites, evaluating it's in vivo and in vitro biological effects, quantifying the concentration of the metabolites and optimising the extraction parameters for the recovery of valuable metabolites will further unlock the potential of kānuka for various industrial and scientific applications. The collaboration with Māori communities and integration of mātauranga Māori into the research ensures that traditional knowledge is honoured and combined with modern scientific methods for sustainable outcomes.

## Supplementary Information

Below is the link to the electronic supplementary material.

Supplementary file1 (DOCX 58 kb)

## Data Availability

The data that support the findings of this study are available on request from corresponding author.

## Abbreviations

ABTS: 2,2′-Azino-bis-(3-ethylbenzothiazoline-6-sulfonic) acid
AchE: Acetylcholinesterase enzyme
AD: Alzheimer's disease
AlCl_3_: Aluminium chloride
DHMDC: 2ʹ,6ʹ-Dihydroxy-4ʹ-methoxy dihydrochalcone
DPPH: 2,2-Diphenyl-1-picrylhydrazyl
FeCl_3_: Ferric chloride
FI: Fragmentation ion
FRAP: Ferric ion-reducing antioxidant power
LC–MS/MS QTOF: Liquid chromatography–mass spectrometry quadrupole time of flight
mg GAE/g dw: Milligram gallic acid equivalent per gram of dry weight
mg QE/g dw: Milligram quercetin equivalent per gram of dry weight
TEAC/g dw: Milligram Trolox equivalent antioxidant capacity per gram of dried weight
PCA: Principal component analysis
RSA: Radical scavenging activity
SWE: Subcritical water extraction
TPC: Total polyphenolic content
TFC: Total flavonoid content

## References

[1] Susawaengsup, C., Jaradrattanapaiboon, A., Sornsakdanuphap, J., Choengpanya, K., Jaradrattanapaiboon, Y., Tongkoom, K., & Bhuyar, P. (2022). Effect of fertilization combined with shading on growth and aromatic constituents of Niamhom (*Strobilanthes nivea* Craib) using an internet of things (IoT) controlled irrigation system. *Horticulturae,**8*(12), 1130. 10.1016/j.fbio.2024.104742

[2] Rao, A., Kumari, S., Laura, J. S., & Dhania, G. (2023). Qualitative phytochemical screening of medicinal plants using different solvent extracts. *Oriental Journal of Chemistry*, *39*(3). 10.13005/ojc/390312

[3] Agostini-Costa, T., & Vieira, R. F., Bizzo, H. R., Silveira, D., & Gimenes, M. A. (2012). Secondary metabolites. *Chromatography and Its Applications,**8*, 131–164. 10.5772/35705

[4] Rasheed, H. A., Rehman, A., Chen, X., Aziz, T., Al-Asmari, F., Alhomrani, M., Alamri, A. S., Cui, H., & Lin, L. (2024). Unveiling the anti-listerial effect of *Citrus bergamia* essential oil: Mechanism of membrane disruption and anti-hemolytic activity. *Food Bioscience,**61*, 104742. 10.1016/j.fbio.2024.104742

[5] Gammatantrawet, N., Nguyễn, C. T., Susawaengsup, C., Ramli, A. N. M., Tongkoom, K., Chatsungnoen, T., Dangtungee, R., & Bhuyar, P. (2024). Phytochemistry of medicinal herbs belongs to asclepiadaceae family for therapeutic applications: A critical review. *Molecular Biotechnology*, *67*. 10.1007/s12033-024-01122-9 10.1007/s12033-024-01122-9 38507018

[6] Shabbir, M. A., Naveed, M., Rehman, S., & Ain NU, Aziz T, Alharbi M, Alsahammari A, Alasmari AF. (2023). Synthesis of iron oxide nanoparticles from *Madhuca indica* plant extract and assessment of their cytotoxic, antioxidant, anti-inflammatory, and anti-diabetic properties via different nanoinformatics approaches. *ACS Omega,**8*, 33358–33366. 10.1021/acsomega.3c02744 37744851 10.1021/acsomega.3c02744 PMC10515396

[7] Waseem, M., Naveed, M., Rehman, S., & Makhdoom, S. I., Aziz, T., Alharbi, M., Alsahammari, A., Alasmari, A. F. (2023). Molecular characterization of spa, hld, fmhA, and lukD genes and computational modeling the multidrug resistance of *Staphylococcus* species through *Callindra harrisii* silver nanoparticles. *ACS Omega,**8*(23), 20920–20936. 10.1021/acsomega.3c01597 37323409 10.1021/acsomega.3c01597 PMC10268295

[8] Aziz, T., Qadir, R., Anwar, F., Naz, S., Nazir, N., Nabi, G., Haiying, C., Lin, L., Alharbi, M., & Alasmari, A. F. (2024). Optimal enzyme-assisted extraction of phenolics from leaves of *Pongamia pinnata* via response surface methodology and artificial neural networking. *Applied Biochemistry and Biotechnology,**196*, 6508–6525. 10.1007/s12010-024-04875-w 38386143 10.1007/s12010-024-04875-w

[9] Essien, S. O., Young, B., & Baroutian, S. (2020). Subcritical water extraction for selective recovery of phenolic bioactives from kānuka leaves. *The Journal of Supercritical Fluids*, *158*. 10.1016/j.supflu.2019.104721

[10] Jamshidi-Kia, F., Lorigooini, Z., & Amini-Khoei, H. (2017). Medicinal plants: Past history and future perspective. *Journal of Herbmed Pharmacology,**7*(1), 1–7. 10.15171/JHP.2018.01

[11] WHO. (2000). General guidelines for methodologies on research and evaluation of traditional medicine. World Health Organization. https://www.who.int/publications/i/item/9789241506090

[12] Adams, O. L. G. (1945). *Maori medicinal plants*. Craig & Lineham.

[13] Brooker, S. G. (1987). *New Zealand medicinal plants*. Heinemann.

[14] Paul, T. (1987). *Nga taonga o te ngahere = Treasures of the forest*. Department of Conservation.

[15] Riley, M. (1994). Māori healing and herbal: New Zealand ethnobotanical sourcebook, Paraparaumu. Viking Sevenseas

[16] Bell, T. W. (1890). Medical notes on New Zealand. *New Zealand Medical Journal,**3*, 129–145.

[17] McGregor, L. W. (1988). Seed of a country: A story of the life and times of John and Janet McGregor, Castle Hill

[18] Andersen, J. C. (2000). *Māori life in Ao-tea*. Cadsonbury Publications.

[19] Goldie, W. H. (1999). *Māori medical lore: Notes on the causes of disease and treatment of the sick among the Māori people of New Zealand, as believed and practised in former times, together with some account of various ancient rites connected with the same*. Kiwi Publishers.

[20] Best, E. (1907). Maori forest lore: Being some account of native lore and woodcraft, as also of many myths, rites, customs and superstitions connected with the flora and fauna of the Tuhoe and Ure-wera District, Auckland Institute.

[21] Chen, Y., & Yen, M. Y., Wu, P. F., Liao, W. T., Huang, T. S., Wen, Z. H., & Wang, H. M. D. (2016). Investigations of kānuka and mānuka essential oils for *in vitro* treatment of disease and cellular inflammation caused by infectious microorganisms. *Journal of Microbiology, Immunology and Infection,**49*(1), 104–111. 10.1016/j.jmii.2013.12.009 10.1016/j.jmii.2013.12.009 24582465

[22] Lis-Balchin, M., Hart, S., & Deans, S. (2000). Pharmacological and antimicrobial studies on different tea-tree oils (*Melaleuca alternifolia*, *Leptospermum scoparium* or Mānuka and *Kunzea ericoides* or Kānuka), originating in Australia and New Zealand. *Phytotherapy Research: An International Journal Devoted to Pharmacological and Toxicological Evaluation of Natural Product Derivatives,**14*(8), 623–629. 10.1002/1099-1573(200012)14:8%3C623::AID-PTR763%3E3.0.CO;2-Z 10.1002/1099-1573(200012)14:8<623::aid-ptr763>3.0.co;2-z 11114000

[23] Maddocks, W., Tayagui, A., Collins, B., Williams, O., & Garrill, A. (2021). Evaluating the effects of kānuka essential oil (*Kunzea ericoides*) grown in different locations in New Zealand on two pathogenic dermatophytes: An *in vitro* study. *American Journal of Essential Oils and Natural Products,**9*(2), 28–33. 10.1016/j.ejon.2009.01.002

[24] Bloor, S. J. (1992). Antiviral phloroglucinols from New Zealand *Kunzea* species. *Journal of Natural Products,**55*(1), 43–47. 10.1021/np50079a006 1318355 10.1021/np50079a006

[25] Essien, S. O., Young, B., & Baroutian, S. (2021). The antibacterial and antiproliferative ability of kānuka, *Kunzea ericoide*s, leaf extracts obtained by subcritical water extraction. *Journal of Chemical Technology & Biotechnology,**96*(5), 1308–1315. 10.1002/jctb.6647

[26] Majid, H., & Silva, F. V. M. (2021). Kānuka bush leaves for Alzheimer’s disease: Improved inhibition of β-secretase enzyme, antioxidant capacity and yield of extracts by ultrasound assisted extraction. *Food and Bioproducts Processing,**128*, 109–120. 10.1016/j.fbp.2021.04.018

[27] Wyatt, R. M., Hodges, L. D., Kalafatis, N., Wright, P. F., Wynne, P. M., & Macrides, T. A. (2005). Phytochemical analysis and biological screening of leaf and twig extracts from *Kunzea ericoides*. *Phytotherapy Research: An International Journal Devoted to Pharmacological and Toxicological Evaluation of Natural Product Derivatives,**19*, 963–970. 10.1002/ptr.1767 10.1002/ptr.1767 16317654

[28] Nobrega, J. R., Silva, D., & de Andrade Junior, F. P., Silva Sousa, P. M., Ramalho de Figueiredo, P. T., Cordeiro, L. V., & Lima, E. D. O. (2021). Antifungal action of alpha-pinene against *Candida* spp. isolated from patients with otomycosis and effects of its association with boric acid. *Natural Product Research,**35*, 6190–6193. 10.1080/14786419.2020.1837803 10.1080/14786419.2020.1837803 33094646

[29] Van Vuuren, S. F., Docrat, Y., Kamatou, G. P. P., & Viljoen, A. M. (2014). Essential oil composition and antimicrobial interactions of understudied tea tree species. *South African Journal of Botany,**92*, 7–14. 10.1016/j.sajb.2014.01.005

[30] Wang, C. Y., Chen, Y. W., & Hou, C. Y. (2019). Antioxidant and antibacterial activity of seven predominant terpenoids. *International Journal of food properties,**22*, 230–238. 10.1080/10942912.2019.1582541

[31] Plaza, M., & Marina, M. L. (2019). Pressurized hot water extraction of bioactives. *TrAC Trends in Analytical Chemistry,**116*, 236–247. 10.1016/j.trac.2019.03.024

[32] Plaza, M., & Turner, C. (2015). Pressurized hot water extraction of bioactives. *TrAC Trends in Analytical Chemistry,**71*, 39–54. 10.1016/j.trac.2015.02.022

[33] Shin, S. B., & Ko, M. J. (2024). Enhanced process for efficient extraction of oxygenated monoterpenes from thyme (*Thymus vulgaris* L.) using subcritical water. *Journal of Industrial and Engineering Chemistry*. 10.1016/j.jiec.2024.07.026

[34] Boller, S., Soldi, C., Marques, M. C. A., Santos, E. P., Cabrini, D. A., Pizzolatti, M. G., Zampronio, A. R., & Otuki, M. F. (2010). Anti-inflammatory effect of crude extract and isolated compounds from *Baccharis illinita* DC in acute skin inflammation. *Journal of Ethnopharmacology,**130*, 262–266. 10.1016/j.jep.2010.05.001 20452414 10.1016/j.jep.2010.05.001

[35] Rasoanaivo, P., Wright, C. W., Willcox, M. L., & Gilbert, B. (2011). Whole plant extracts versus single compounds for the treatment of malaria: Synergy and positive interactions. *Malaria journal,**10*, 1–12. 10.1186/1475-2875-10-S1-S4 21411015 10.1186/1475-2875-10-S1-S4 PMC3059462

[36] Hocart, C. H. (2010). Mass spectrometry: An essential tool for trace identification and quantification. *Comprehensive Natural Products II,**1*, 327–388. 10.1016/B978-008045382-8.00187-8

[37] Attard, E. (2013). A rapid microtitre plate Folin-Ciocalteu method for the assessment of polyphenols. *Open Life Sciences,**8*, 48–53. 10.2478/s11535-012-0107-3

[38] Zhang, Q., Zhang, J., Shen, J., Silva, A., Dennis, D. A., & Barrow, C. J. (2006). A simple 96-well microplate method for estimation of total polyphenol content in seaweeds. *Journal of applied phycology,**18*, 445–450. 10.1007/s10811-006-9048-4

[39] Susawaengsup, C., Choengpanya, K., Sornsakdanuphap, J., Tabtimmai, L., Chaiharn, M., & Bhuyar, P. (2024). Phytochemical and pharmacological properties of a traditional herb, *Strobilanthes Cusia* (Nees) Kuntze. *Molecular Biotechnology,**66*, 2860–2871. 10.1007/s12033-023-00897-7 37773314 10.1007/s12033-023-00897-7

[40] Lawag, I. L., Nolden, E. S., Schaper, A. A., Lim, L. Y., & Locher, C. (2023). A modified Folin-Ciocalteu assay for the determination of total phenolics content in honey. *Applied Sciences,**13*, 2135. 10.3390/app13042135

[41] Sánchez-Rangel, J. C., Benavides, J., Heredia, J. B., Cisneros-Zevallos, L., & Jacobo-Velázquez, D. A. (2013). The Folin-Ciocalteu assay revisited: Improvement of its specificity for total phenolic content determination. *Analytical methods,**5*, 5990–5999. 10.1039/c3ay41125g

[42] Herald, T. J., Gadgil, P., & Tilley, M. (2012). High-throughput micro plate assays for screening flavonoid content and DPPH-scavenging activity in sorghum bran and flour. *Journal of the Science of Food and Agriculture,**92*, 2326–2331. 10.1002/jsfa.5633 22419130 10.1002/jsfa.5633

[43] Re, R., Pellegrini, N., Proteggente, A., Pannala, A., Yang, M., & Rice-Evans, C. (1999). Antioxidant activity applying an improved ABTS radical cation decolorization assay. *Free radical biology and medicine,**26*, 1231–1237. 10.1016/S0891-5849(98)00315-3 10381194 10.1016/s0891-5849(98)00315-3

[44] Kumar, S., Chandra, P., Bajpai, V., Singh, A., Srivastava, M., Mishra, D., & Kumar, B. (2015). Rapid qualitative and quantitative analysis of bioactive compounds from Phyllanthus amarus using LC/MS/MS techniques. *Industrial Crops and Products,**69*, 143–152. 10.1016/j.indcrop.2015.02.012

[45] Reddy, A., Norris, D. F., Momeni, S. S., Waldo, B., & Ruby, J. D. (2016). The pH of beverages in the United States. *The Journal of the American Dental Association,**147*, 255–263. 10.1016/j.adaj.2015.10.019 26653863 10.1016/j.adaj.2015.10.019 PMC4808596

[46] Arabshahi-D, S., Vishalakshi Devi, D., & Urooj, A. (2007). Evaluation of antioxidant activity of some plant extracts and their heat, pH and storage stability. *Food Chemistry,**100*, 1100–1105. 10.1016/j.foodchem.2005.11.014

[47] Bayliak, M. M., Burdyliuk, N. I., & Lushchak, V. I. (2016). Effects of pH on antioxidant and prooxidant properties of common medicinal herbs. *Open Life Sciences,**11*, 298–307. 10.1515/biol-2016-0040

[48] Kadir, D. H. (2021). Statistical evaluation of main extraction parameters in twenty plant extracts for obtaining their optimum total phenolic content and its relation to antioxidant and antibacterial activities. *Food Science & Nutrition,**9*, 3491–3499. 10.1002/fsn3.2288 34262709 10.1002/fsn3.2288 PMC8269642

[49] Friedman, M., & Jürgens, H. S. (2000). Effect of pH on the stability of plant phenolic compounds. *Journal of Agricultural and Food Chemistry,**48*, 2101–2110. 10.1021/jf990489j 10888506 10.1021/jf990489j

[50] Doughari, J. (2006). Antimicrobial activity of *Tamarindus indica* Linn. *Tropical Journal of Pharmaceutical Research,**5*, 597–603. 10.4314/tjpr.v5i2.14637

[51] Rangsriwong, P., Rangkadilok, N., Satayavivad, J., Goto, M., & Shotipruk, A. (2009). Subcritical water extraction of polyphenolic compounds from *Terminalia chebula* Retz. fruits. *Separation and Purification Technology,**66*, 51–56. 10.1016/j.seppur.2008.11.023

[52] Fabian, C., Tran-Thi, N. Y., Kasim, N. S., & Ju, Y. H. (2010). Release of phenolic acids from defatted rice bran by subcritical water treatment. *Journal of the science of food and agriculture,**90*, 2576–2581. 10.1002/jsfa.4123 20687234 10.1002/jsfa.4123

[53] He, L., Zhang, X., Xu, H., Xu, C., Yuan, F., Knez, Ž, Novak, Z., & Gao, Y. (2012). Subcritical water extraction of phenolic compounds from pomegranate (*Punica granatum* L.) seed residues and investigation into their antioxidant activities with HPLC–ABTS+ assay. *Food and Bioproducts Processing,**90*, 215–223. 10.1016/j.fbp.2011.03.003

[54] Korus, A. (2011). Effect of preliminary processing, method of drying and storage temperature on the level of antioxidants in kale (*Brassica oleracea* L. var. acephala) leaves. *LWT - Food Science and Technology,**44*, 1711–1716. 10.1016/j.lwt.2011.03.014

[55] Ullah, S., Khan, R., Hussain, A., Ullah, A., & Khaliq-Ur-Rehman, K.-U.-R. (2017). Effects of temperature and storage on the antioxidant potential, polyphenols and vitamin-C contents of *Azadirachta Indica* leave aqueous extract. *Pakistan journal of pharmaceutical sciences,**30*, 1665–1669. 29084688

[56] Baltacioğlu, C. E. M., Velioğlu, S., & Karacabey, E. (2011). Changes in total phenolic and flavonoid contents of rowanberry fruit during postharvest storage. *Journal of food quality,**34*, 278–283. 10.1111/j.1745-4557.2011.00389.x

[57] Putra, N. R., Rizkiyah, D. N., Idham, Z., Veza, I., Qomariyah, L., & Yunus, M. A. C. (2024). Optimization and modelling in flavonoid and phenolic compounds recovery from peanut skin by subcritical water. *Biomass Conversion and Biorefinery,**14*, 12299–12309. 10.1007/s13399-022-03263-w

[58] Ko, M. J., Cheigh, C. I., & Chung, M. S. (2014). Relationship analysis between flavonoids structure and subcritical water extraction (SWE). *Food Chemistry,**143*, 147–155. 10.1016/j.foodchem.2013.07.104 24054224 10.1016/j.foodchem.2013.07.104

[59] Floegel, A., Kim, D. O., Chung, S. J., Koo, S. I., & Chun, O. K. (2011). Comparison of ABTS/DPPH assays to measure antioxidant capacity in popular antioxidant-rich US foods. *Journal of Food Composition and Analysis,**24*, 1043–1048. 10.1016/j.jfca.2011.01.008

[60] Untea, A., Lupu, A., Saracila, M., & Panaite, T. (2018). Comparison of ABTS, DPPH, phosphomolybdenum assays for estimating antioxidant activity and phenolic compounds in five different plant extracts. *Bulletin UASVM Animal Science and Biotechnologies*. 10.15835/buasvmcn-asb:2018.0009

[61] Yoshino, M., & Murakami, K. (1998). Interaction of iron with polyphenolic compounds: Application to antioxidant characterization. *Analytical Biochemistry,**257*, 40–44. 10.1006/abio.1997.2522 9512770 10.1006/abio.1997.2522

[62] Dudonné, S., Vitrac, X., Coutière, P., Woillez, M., & Mérillon, J.-M. (2009). Comparative study of antioxidant properties and total phenolic content of 30 plant extracts of industrial interest using DPPH, ABTS, FRAP, SOD, and ORAC Assays. *Journal of Agricultural and Food Chemistry,**57*, 1768–1774. 10.1021/jf803011r 19199445 10.1021/jf803011r

[63] Deng, Y., & Lu, S. (2017). Biosynthesis and regulation of phenylpropanoids in plants. *Critical Reviews in Plant Sciences,**36*, 257–290. 10.1080/07352689.2017.1402852

[64] Ahad, A., Ganai, A. A., Mujeeb, M., & Siddiqui, W. A. (2014). Ellagic acid, an NF-κB inhibitor, ameliorates renal function in experimental diabetic nephropathy. *Chemico-Biological Interactions,**219*, 64–75. 10.1016/j.cbi.2014.05.011 24877639 10.1016/j.cbi.2014.05.011

[65] Amin, M. M., & Arbid, M. S. (2017). Estimation of ellagic acid and/or repaglinide effects on insulin signaling, oxidative stress, and inflammatory mediators of liver, pancreas, adipose tissue, and brain in insulin resistant/type 2 diabetic rats. *Applied Physiology, Nutrition, and Metabolism,**42*, 181–192. 10.1139/apnm-2016-0429 28092161 10.1139/apnm-2016-0429

[66] Jiménez-Ferrer, E., Alarcón-Alonso, J., Aguilar-Rojas, A., Zamilpa, A., Jiménez-Ferrer, C. I., Tortoriello, J., & Herrera-Ruiz, M. (2012). Diuretic effect of compounds from *Hibiscus sabdariffa* by modulation of the aldosterone activity. *Planta Medica,**78*, 1893–1898. 10.1055/s-0032-1327864 23150077 10.1055/s-0032-1327864

[67] Wahyudiono, W., Maeda, S., Machmudah, S., Sato, K., Kanda, H., & Goto, M. (2022). Extraction of procyanidin B2 from apple peel using subcritical water. *ASEAN Engineering Journal,**12*, 135–141.

[68] Ko, M. J., Cheigh, C. I., & Chung, M. S. (2014). Optimization of subcritical water extraction of flavanols from green tea leaves. *Journal of Agricultural and Food Chemistry,**62*, 6828–6833. 10.1021/jf5012006 24918863 10.1021/jf5012006

[69] Hümmer, W., & Schreier, P. (2008). Analysis of proanthocyanidins. *Molecular Nutrition & Food Research,**52*, 1381–1398. 10.1002/mnfr.200700463 19065593 10.1002/mnfr.200700463

[70] Dallas, C., Ricardo-da-Silva, J., & Laureano, O. (1995). Degradation of oligomeric procyanidins and anthocyanins in a Tinta Roriz red wine during maturation. *Vitis,**34*, 51–56. 10.5073/vitis.1995.34.51-56

[71] Babu, P. V. A., & Liu, D. (2009). Flavonoids and cardiovascular health. *Complementary and alternative therapies and the aging population* (pp. 371–392). Elsevier.

[72] Ko, M. J., Nam, H. H., & Chung, M. S. (2020). Subcritical water extraction of bioactive compounds from *Orostachys japonicus* A. Berger (Crassulaceae). *Scientific Reports,**10*, 10890. 10.1038/s41598-020-67508-2 32616865 10.1038/s41598-020-67508-2 PMC7331711

[73] Cheigh, C. I., Yoo, S. Y., Ko, M. J., Chang, P. S., & Chung, M. S. (2015). Extraction characteristics of subcritical water depending on the number of hydroxyl group in flavonols. *Food Chemistry,**168*, 21–26. 10.1016/j.foodchem.2014.07.047 25172678 10.1016/j.foodchem.2014.07.047

[74] Cuyckens, F., & Claeys, M. (2004). Mass spectrometry in the structural analysis of flavonoids. *Journal of Mass Spectrometry,**39*, 1–15. 10.1002/jms.585 14760608 10.1002/jms.585

[75] Gonçalves, A. E., Malheiros, Â., Casarin, C. A., de França, L., Palomino-Salcedo, D. L., Ferreira, L. L. G., Andricopulo, A. D., Dávila-Rodríguez, M. J., Lacava, L. C., de Oliveira, A. S., & de Souza, M. M. (2021). 2’,6’-Dihydroxy-4’-methoxy dihydrochalcone improves the cognitive impairment of Alzheimer’s disease: A structure-activity relationship study. *Current Topics in Medicinal Chemistry,**21*, 1167–1185. 10.2174/1568026621666210701114034 34218788 10.2174/1568026621666210701114034

[76] Jin, C. H., Shin, E. J., Park, J. B., Jang, C. G., Li, Z., Kim, M. S., Koo, K. H., Yoon, H. J., Park, S. J., & Choi, W. C. (2009). Fustin flavonoid attenuates β-amyloid (1–42)-induced learning impairment. *Journal of Neuroscience Research,**87*, 3658–3670. 10.1002/jnr.22159 19533734 10.1002/jnr.22159

[77] Kim, J. B., Han, A. R., Park, E. Y., Kim, J. Y., Cho, W., Lee, J., Seo, E. K., & Lee, K. T. (2007). Inhibition of LPS-Induced iNOS, COX-2 and cytokines expression by poncirin through the NF-kB inactivation in RAW 264.7 macrophage cells. *Biological and Pharmaceutical Bulletin,**30*, 2345–2351. 10.1248/bpb.30.2345 18057724 10.1248/bpb.30.2345

[78] Yoon, H. Y., Won, Y. Y., & Chung, Y. S. (2012). Poncirin prevents bone loss in glucocorticoid-induced osteoporosis *in vivo* and *in vitro*. *Journal of bone and mineral metabolism,**30*, 509–516. 10.1007/s00774-012-0350-8 22407507 10.1007/s00774-012-0350-8

[79] Ramchundar, N., & Nlooto, M. (2020). A comparative qualitative study of the types of traditional treatment of fractures by traditional health practitioners in Kwazulu-Natal, South Africa and the North Island of New Zealand: A survey-based study. *International Journal,**1*, 2.

[80] Das, S., Rohman, M. A., & Singha Roy, A. (2018). Exploring the non-covalent binding behaviours of 7-hydroxyflavone and 3-hydroxyflavone with hen egg white lysozyme: Multi-spectroscopic and molecular docking perspectives. *Journal of Photochemistry and Photobiology B: Biology,**180*, 25–38. 10.1016/j.jphotobiol.2018.01.021 29413699 10.1016/j.jphotobiol.2018.01.021

[81] Parthiban, A., Sachithanandam, V., Lalitha, P., Elumalai, D., Asha, R. N., Jeyakumar, T. C., Muthukumaran, J., Jain, M., Jayabal, K., Mageswaran, T., Sridhar, R., Purvaja, R., & Ramesh, R. (2023). Isolation and biological evaluation 7-hydroxy flavone from *Avicennia officinalis* L: Insights from extensive in vitro, DFT, molecular docking and molecular dynamics simulation studies. *Journal of Biomolecular Structure & Dynamics,**41*, 2848–2860. 10.1080/07391102.2022.2039771 35193476 10.1080/07391102.2022.2039771

[82] Nakagawa, M. (1975). Chemical components and taste of green tea. *Japan Agricultural Research Quarterly,**9*, 156–160.

[83] Narukawa, M., Kimata, H., Noga, C., & Watanabe, T. (2010). Taste characterisation of green tea catechins. *International Journal of Food Science & Technology,**45*, 1579–1585. 10.1111/j.1365-2621.2010.02304.x

[84] Balentine, D. A., Wiseman, S. A., & Bouwens, L. C. (1997). The chemistry of tea flavonoids. *Critical Reviews in Food Science & Nutrition,**37*, 693–704. 10.1080/10408399709527797 9447270 10.1080/10408399709527797

[85] Ni, K., Guo, J., Bu, B., Pan, Y., Li, J., Liu, L., Luo, M., & Deng, L. (2021). Naringin as a plant-derived bitter tastant promotes proliferation of cultured human airway epithelial cells via activation of TAS2R signaling. *Phytomedicine,**84*, 153491. 10.1016/j.phymed.2021.153491 33601237 10.1016/j.phymed.2021.153491

[86] Wink, M. (2015). Modes of action of herbal medicines and plant secondary metabolites. *Medicines (Basel, Switzerland),**2*, 251–286. 10.3390/medicines2030251 28930211 10.3390/medicines2030251 PMC5456217

[87] Baell, J. B., & Nissink, J. W. M. (2018). Seven year itch: Pan-Assay Interference Compounds (PAINS) in 2017-utility and limitations. *ACS Chemical Biology,**13*, 36–44. 10.1021/acschembio.7b00903 29202222 10.1021/acschembio.7b00903 PMC5778390

[88] Baell, J. B. (2016). Feeling nature’s PAINS: Natural products, natural product drugs, and pan assay interference compounds (PAINS). *Journal of Natural Products,**79*, 616–628. 10.1021/acs.jnatprod.5b00947 26900761 10.1021/acs.jnatprod.5b00947

[89] Jagtap, S., Meganathan, K., Wagh, V., Winkler, J., Hescheler, J., & Sachinidis, A. (2009). Chemoprotective mechanism of the natural compounds, epigallocatechin-3-O-gallate, quercetin and curcumin against cancer and cardiovascular diseases. *Current medicinal chemistry,**16*, 1451–1462. 10.2174/092986709787909578 19355899 10.2174/092986709787909578

[90] Sinosaki, N. B., Tonin, A. P., Ribeiro, M. A., Poliseli, C. B., Roberto, S. B., Silveira, R. D., Visentainer, J. V., Santos, O. O., & Meurer, E. C. (2020). Structural study of phenolic acids by triple quadrupole mass spectrometry with electrospray ionization in negative mode and H/D isotopic exchange. *Journal of the Brazilian Chemical Society,**31*, 402–408.

[91] Shi, J., Gao, X., Zhang, A., Qin, X., & Du, G. (2022). Characterization of multiple chemical components of GuiLingJi by UHPLC-MS and 1H NMR analysis. *Journal of Pharmaceutical Analysis,**12*, 460–469. 10.1016/j.jpha.2021.09.013 35811626 10.1016/j.jpha.2021.09.013 PMC9257439

[92] Mok, H. W., Ko, M. J., Choi, H. J., & Chung, M. S. (2022). Extraction of chlorogenic acids from hibiscus (*Hibiscus syriacus* L.) by subcritical-water. *Journal of Industrial and Engineering Chemistry,**111*, 255–262. 10.1016/j.jiec.2022.04.005

[93] Pearson, J. L., Lee, S., Suresh, H., Low, M., Nang, M., Singh, S., Lamin, F., Kazzem, M., Sullivan, S., & Khoo, C. S. (2014). The liquid chromatographic determination of chlorogenic and caffeic acids in Xu Duan (*Dipsacus asperoides*) raw herb. *International Scholarly Research Notices*. 10.1155/2014/968314

[94] Gabaston, J., Leborgne, C., Valls, J., Renouf, E., Richard, T., Waffo-Teguo, P., & Mérillon, J. M. (2018). Subcritical water extraction of stilbenes from grapevine by-products: A new green chemistry approach. *Industrial Crops and Products,**126*, 272–279. 10.1016/j.indcrop.2018.10.020

[95] Tian, J., Jin, L., Liu, H., & Hua, Z. (2023). Stilbenes: A promising small molecule modulator for epigenetic regulation in human diseases. *Frontiers in pharmacology,**14*, 1326682. 10.3389/fphar.2023.1326682 38155902 10.3389/fphar.2023.1326682 PMC10754530

[96] Özkaynak Kanmaz, E., & Ova, G. (2013). The effective parameters for subcritical water extraction of SDG lignan from flaxseed (*Linum usitatissimum* L.) using accelerated solvent extractor. *European Food Research and Technology,**237*, 159–166. 10.1007/s00217-013-1974-1

