# Supplementary material for: Subcritical Water Extraction of Kānuka (Kunzea ericoides): A Qualitative Analysis of Bioactive Profile Based on Antioxidant Properties
Source: Mol Biotechnol. 2025 Feb 24;68(2):669–92. doi: 10.1007/s12033-025-01399-4 (PMC12967634; doi:10.1007/s12033-025-01399-4)
Supplement: Supplementary file 1 — Supplementary file1 (DOCX 58 kb) [file 12033_2025_1399_MOESM1_ESM.docx]

**Subcritical Water Extraction of Kānuka *(Kunzea ericoides)*: Qualitative Analysis of Bioactive Profile Based on Antioxidant Properties**

Indhuja Devadass *^a^*, Simon Swift *^b^*, Sree Sreebhavan*^c^*, Saeid Baroutian *^a,d,*^*

*^a^ Department of Chemical and Materials Engineering, The University of Auckland, Auckland 1010, New Zealand*

*^b^ School of Medical Sciences, Faculty of Medical and Health Sciences, The University of Auckland, Auckland 1010, New Zealand*

*^c^ Auckland Cancer Society Research Centre, The University of Auckland, Auckland 1010, New Zealand*

*^d^ Circular Innovations (CIRCUIT) Research Centre, The University of Auckland, Auckland 1010, New Zealand*

**S1.** Bioactive compounds of kānuka subcritical water extract obtained at 180℃, 200℃ and 220℃ at 5 min, 10 min and 15 min time interval and identified using LC-MS/MS QTOF-ESI (positive and negative ionisation)

| **Compounds** | **Molecular formula** | **Theoritical mass (g/mol)** | **Observed mass (g/mol)** | **Retention time (min)** | **precursor ion (m/z)** | **Error difference (ppm)** | **ID Score** | **Species** |
| --- | --- | --- | --- | --- | --- | --- | --- | --- |
| **Flavonoids** |  |  |  |  |  |  |  |  |
| Gossypetin | C_15_ H_10_ O_8_ | 318.038 | 318.036 | 4.221 | 319.044 | -4.2 | 94.21 | (M+H)+ |
| 7-Hydroxyflavone | C_15_ H_10_ O_3_ | 238.063 | 238.062 | 9.61 | 240.073 | -3.26 | 83.72 | (M+H)+ |
| Chrysoeriol 7-O-glucoside | C_22_ H_22_ O_11_ | 462.116 | 462.114 | 9.747 | 463.121 | -4.78 | 84.4 | (M+H)+ |
| Tricin | C_17_ H_14_ O_7_ | 330.074 | 330.074 | 15.672 | 331.082 | 0.05 | 88.91 | (M+H)+ |
| 4',5,6,7-Tetramethoxyflavone | C_19_ H_18_ O_6_ | 342.11 | 342.11 | 10.714 | 343.115 | -1.29 | 68.57 | (M+H)+ |
| Baicalein 5,6,7-trimethyl ether | C_18_ H_16_ O_5_ | 312.1 | 312.101 | 11.93 | 313.108 | 2.24 | 64.03 | (M+H)+ |
| Pinobanksin 3-O-acetate | C_17_ H_14_ O_6_ | 314.079 | 314.078 | 17.584 | 315.084 | -4.12 | 68.59 | (M+H)+ |
| Pinocembrin | C_15_ H_12_ O_4_ | 256.074 | 256.073 | 7.936 | 257.081 | -3.55 | 61.23 | (M+H)+ |
| (+)-Catechin | C_15_ H_14_ O_6_ | 290.079 | 290.078 | 2.929 | 291.085 | -3.61 | 76.53 | (M+H)+ |
| Baicalin | C_21_ H_18_ O_11_ | 446.085 | 446.084 | 8.65 | 447.092 | -1.17 | 73.93 | (M+H)+ |
| Afzelechin | C_15_ H_14_ O_5_ | 274.084 | 274.084 | 9.165 | 275.091 | 0.37 | 83.39 | (M+H)+ |
| Apigenin 7-O-[beta-D-apiosyl-(1- >2)-beta-D-glucoside | C_26_ H_28_ O_14_ | 564.148 | 564.148 | 6.955 | 565.156 | -0.66 | 60.76 | (M+H)+ |
| Vitexin | C_21_ H_20_ O_10_ | 432.106 | 432.106 | 8.91 | 434.113 | 0.2 | 80.12 | (M+H)+ |
| Apigenin 7-O-[beta-D-apiosyl-(1- >2)-beta-D-glucoside] | C_26_ H_28_ O_14_ | 564.148 | 564.149 | 3.002 | 565.155 | 1.17 | 64.54 | (M+H)+ |
| Quercetin 3'-O-glucuronide | C_21_ H_18_ O_13_ | 478.075 | 478.075 | 10.365 | 479.082 | 0.42 | 74.88 | (M+H)+ |
| 5,7-Dimethoxyflavone | C_17_ H_14_ O_4_ | 282.089 | 282.089 | 14.351 | 283.096 | -0.23 | 82.69 | (M+H)+ |
| 2',6'-Dihydroxy-4'- methoxydihydrochalcone | C_16_ H_16_ O_4_ | 272.105 | 272.104 | 9.842 | 273.111 | -2.77 | 81.73 | (M+H)+ |

| **Compounds** | **Molecular formula** | **Theoritical mass (g/mol)** | **Observed mass (g/mol)** | **Retention time (min)** | **precursor ion (m/z)** | **Error difference (ppm)** | **ID Score** | **Species** |
| --- | --- | --- | --- | --- | --- | --- | --- | --- |
| **Flavonoids** |  |  |  |  |  |  |  |  |
| Garbanzol | C_15_ H_12_ O_5_ | 272.069 | 272.069 | 5.361 | 273.075 | 0.23 | 71.88 | (M+H)+ |
| Quercetin 3-O-glucosyl-xyloside | C_26_ H_28_ O_16_ | 596.138 | 596.137 | 12.935 | 595.13 | -0.56 | 69.38 | (M-H)- |
| Gardenin B | C_19_ H_18_ O_7_ | 358.106 | 358.106 | 16.807 | 359.115 | 2.76 | 65.94 | (M+H)+ |
| Tectochrysin | C_16_ H_12_ O_4_ | 268.074 | 268.073 | 4.038 | 269.08 | -3.76 | 77.35 | (M+H)+ |
| p-Coumaric acid 4-O-glucoside | C_15_ H_18_ O_8_ | 326.1 | 326.1 | 8.961 | 325.092 | -0.68 | 74.04 | (M-H)- |
| (+)-Gallocatechin | C_15_ H_14_ O_7_ | 306.074 | 306.074 | 4.324 | 305.067 | -0.42 | 88.51 | (M-H)- |
| Naringin | C_27_ H_32_ O_14_ | 580.179 | 580.179 | 13.689 | 579.172 | 0.34 | 72.88 | (M-H)- |
| Fustin | C_15_ H_12_ O_6_ | 288.063 | 288.063 | 8.351 | 287.056 | -1.33 | 79.98 | (M-H)- |
| Phellamurin | C_26_ H_30_ O_11_ | 518.179 | 518.179 | 15.585 | 517.173 | -0.25 | 62.78 | (M-H)- |
| Scutellarein | C_15_ H_10_ O_6_ | 286.048 | 286.047 | 2.01 | 286.045 | -2.82 | 78.18 | (M-H)- |
| Neohesperidin | C_28_ H_34_ O_15_ | 610.19 | 610.191 | 5.177 | 610.187 | 1.51 | 92.95 | (M-H)- |
| Hispidulin | C_16_ H_12_ O_6_ | 300.063 | 300.063 | 13.919 | 300.056 | -3.13 | 71.66 | (M-H)- |
| Poncirin | C_28_ H_34_ O_14_ | 594.195 | 594.196 | 15.619 | 593.189 | 1.5 | 85.89 | (M-H)- |
| Morin | C_15_ H_10_ O_7_ | 302.043 | 302.041 | 11.856 | 302.037 | -4.78 | 93.08 | (M-H)- |
| **Stillbenes** |  |  |  |  |  |  |  |  |
| Resveratrol | C_14_ H_12_ O_3_ | 228.079 | 228.079 | 19.535 | 229.085 | -0.63 | 70.68 | (M+H)+ |
| Pinosylvin | C_14_ H_12_ O_2_ | 212.084 | 212.083 | 9.658 | 211.076 | -3.23 | 78.72 | (M-H)- |
| Astringin | C_20_ H_22_ O_9_ | 406.126 | 406.125 | 9.032 | 405.119 | -3.44 | 64.57 | (M-H)- |

**S1** continue

| **Compounds** | **Molecular formula** | **Theoritical mass (g/mol)** | **Observed mass (g/mol)** | **Retention time (min)** | **precursor ion (m/z)** | **Error difference (ppm)** | **ID Score** | **Species** |
| --- | --- | --- | --- | --- | --- | --- | --- | --- |
| **Phenolic acids** |  |  |  |  |  |  |  |  |
| 2-Hydroxybenzoic acid | C_7_ H_6_ O_3_ | 138.032 | 138.031 | 3.634 | 139.038 | -3.74 | 83.16 | (M+H)+ |
| Ellagic acid | C_14_ H_6_ O_8_ | 302.006 | 302.005 | 1.791 | 303.012 | -4.24 | 93.9 | (M+H)+ |
| Caffeic acid | C_9_ H_8_ O_4_ | 180.042 | 180.042 | 3.714 | 181.05 | 0.83 | 63.51 | (M+H)+ |
| Chlorogenic acid | C_16_ H_18_ O_9_ | 354.095 | 354.095 | 2.106 | 355.102 | 0.63 | 81.14 | (M+H)+ |
| Isochlorogenic acid b | C_25_ H_24_ O_12_ | 516.127 | 516.126 | 8.459 | 517.132 | -2.1 | 71.06 | (M+H)+ |
| Ferulate | C_10_H_10_O_4_ | 194.058 | 194.058 | 5.24 | 195.065 | 1.14 | 76.15 | (M+H)+ |
| 5-Hydroxyferulic acid | C_10_ H_10_ O_5_ | 210.053 | 210.052 | 2.105 | 209.044 | -4.22 | 63.11 | (M-H)- |
| Trans-Cinnamate (Cinnamic acid) | C_9_ H_8_ O_2_ | 148.052 | 148.052 | 12.676 | 149.059 | -3.55 | 55.77 | (M+H)+ |
| 3-p-Coumaroylquinic acid | C_16_ H_18_ O_8_ | 338.1 | 338.1 | 1.695 | 337.093 | -0.4 | 80.37 | (M-H)- |
| Sinapic acid | C_11_ H_12_ O_5_ | 224.069 | 224.068 | 9.364 | 223.06 | -4.18 | 70.22 | (M-H)- |
| 3-Feruloylquinic acid | C_17_ H_20_ O_9_ | 368.111 | 368.11 | 4.318 | 367.103 | -2.63 | 80.01 | (M-H)- |
| Quinic acid | C_7_ H_12_ O_6_ | 192.063 | 192.064 | 3.932 | 191.056 | 1.91 | 85.15 | (M-H)- |
| Gallic acid | C_7_ H_6_ O_5_ | 170.022 | 170.021 | 1.798 | 169.014 | -4.25 | 83.69 | (M-H)- |
| 4-Sinapoylquinic acid | C_18_ H_22_ O_10_ | 398.121 | 398.121 | 5.91 | 397.114 | -0.56 | 75.49 | (M-H)- |
| **Monolignols** |  |  |  |  |  |  |  |  |
| Matairesinol | C_20_ H_22_ O_6_ | 358.144 | 358.143 | 12.213 | 359.153 | 3.98 | 62.62 | (M+H)+ |
| Schisandrin C | C_22_ H_24_ O_6_ | 384.157 | 384.156 | 21.693 | 385.162 | -3.67 | 68.41 | (M+H)+ |

**S1** continue

| **S1** continue | | | | | | | | |
| --- | --- | --- | --- | --- | --- | --- | --- | --- |
| **Compounds** | **Molecular formula** | **Theoretical mass (g/mol)** | **Observed mass (g/mol)** | **Retention time (min)** | **precursor ion (m/z)** | **Error difference (ppm)** | **ID Score** | **Species** |
| **Coumarins** |  |  |  |  |  |  |  |  |
| Daphnetin | C_9_ H_6_ O_4_ | 178.027 | 178.026 | 15.209 | 179.034 | -2.84 | 77.31 | (M+H)+ |
| Isopimpinellin | C_13_ H_10_ O_5_ | 246.053 | 246.052 | 16.853 | 247.06 | -3.2 | 76.78 | (M+H)+ |
| Scopoletin | C_10_ H_8_ O_4_ | 192.042 | 192.042 | 10.797 | 194.052 | 0.1 | 86.09 | (M+H)+ |
| **Others** |  |  |  |  |  |  |  |  |
| Coniferyl aldehyde | C_10_ H_10_ O_3_ | 178.063 | 178.063 | 8.427 | 179.07 | 0.97 | 68.76 | (M+H)+ |
| Piceatannol | C_14_ H_12_ O_4_ | 244.074 | 244.074 | 2.818 | 245.082 | 0.61 | 58.06 | (M+H)+ |
| 6-Gingerol | C_17_ H_26_ O_4_ | 294.183 | 294.183 | 26.398 | 293.176 | -1.17 | 84.59 | (M-H)- |
| 4-Coumarate | C_9_ H_8_ O_3_ | 164.047 | 164.046 | 19.574 | 165.054 | -5.89 | 78.89 | (M+H)+ |
| 4-Hydroxybenzaldehyde | C_7_ H_6_ O_2_ | 122.037 | 122.037 | 3.409 | 123.043 | -1.11 | 79.16 | (M+H)+ |
| Silandrin | C_25_ H_22_ O_9_ | 466.126 | 466.125 | 1.71 | 465.118 | -2.32 | 57.07 | (M-H)- |
| Silychristin | C_25_ H_22_ O_10_ | 482.121 | 482.121 | 10.169 | 481.114 | -1.38 | 69.78 | (M-H)- |
| Todolactol A | C_20_ H_24_ O_7_ | 376.152 | 376.152 | 15.52 | 375.142 | -1.76 | 68.56 | (M-H)- |
| 2-Hydroxy-2-phenylacetic acid | C_8_ H_8_ O_3_ | 152.047 | 152.047 | 7.286 | 151.04 | -3.43 | 56.57 | (M-H)- |
| Dihydroconiferyl alcohol | C_10_ H_14_ O_3_ | 182.094 | 182.094 | 11.51 | 181.086 | -3.47 | 84.89 | (M-H)- |
| 4-Coumaryl alcohol | C_9_ H_10_ O_2_ | 150.068 | 150.069 | 4.348 | 149.061 | 3.88 | 77.45 | (M-H)- |
| Verbascoside | C_29_ H_36_ O_15_ | 624.205 | 624.208 | 17.417 | 623.2 | 3.33 | 87.61 | (M-H)- |

**S2**. Fragmentation pattern of bioactive compounds identified in kānuka subcritical water extract obtained at different temperatures (170℃, 180℃, 200℃ and 220℃) and time (5 min, 10 min and 15 min)

| **Compounds** | **Species** | **Fragmentation ion (m/z)** | **Precursor ion** |
| --- | --- | --- | --- |
| (+)-Gallocatechin | (M-H)- | 59.0136; 96.9592; 225.1096 | 305.0631 |
| Betagarin | (M-H)- | 93.0329; 107.0495; 119.0502; 124.0161; 135.0450; 137.0600; 145.0282; 150.0319 | 163.0392 |
| Morin | (M-H)- | 121.0258; 151.0010; 178.9971; 261.1501 | 301.0327 |
| Dihydroconiferyl alcohol | (M-H)- | 113.0968; 166.0619 | 181.0859 |
| Pinosylvin | (M-H)- | 115.4188; 169.0668 | 211.0733 |
| Resveratrol | (M-H)- | 55.0182; 130.0413; 199.0740 | 227.0688 |
| Dihydroferulic acid 4-O-glucuronide | (M-H)- | 59.0120; 121.0288; 175.0266; 175.0266; 175.0266 | 371.0974 |
| 4-Coumarate | (M-H)- | 63.0193; 93.0320; 135.0431 | 163.0377 |
| 5-Hydroxymethylfurfural | (M-H)- | 53.0397; 69.0354; 79.0200; 97.0294; 107.0141 | 125.0235 |
| Gallic acid | (M-H)- | 79.0186; 107.0138; 125.0232 | 169.0110 |
| Chrysoeriol 7-O-glucoside | (M-H)- | 285.0392; 315.0491 | 461.1051 |
| (6E)-8-Oxogeranial | (M-H)- | 59.0152; 81.0344; 123.0804 | 165.0914 |
| Dihydrocaffeic acid 3-O-glucuronide | (M-H)- | 61.1286; 121.0303; 177.0443; 232.0643; 249.0609; 289.0735 | 371.0928 |
| Azaleatin | (M-H)- | 121.0255; 165.0192; 193.0115 | 315.0499 |
| Quinic acid | (M-H)- | 85.0299; 93.0348; 127.0370 | 191.0549 |
| Strobopinin | (M-H)- | 121.0262; 165.0170; 225.0922 | 269.0807 |
| Pinocembrin | (M-H)- | 63.0231; 83.0137; 107.0136; 151.0019; 168.9878; 213.0525 | 255.0637 |
| Scutellarein | (M-H)- | 75.9179; 163.6842 | 285.0399 |
| 3,4-Dihydroxystyrene | (M-H)- | 92.0282 | 135.0422 |
| Coniferyl alcohol | (M-H)- | 135.0799; 137.0268 | 179.0694 |

**Table S2**. Continue.

| **Compounds** | **Species** | **Fragmentation ion (m/z)** | **Precursor ion** |
| --- | --- | --- | --- |
| Pinosylvin | (M-H)- | 131.3907; 169.0639 | 211.0726 |
| Tectochrysin | (M-H)- | 73.9611; 113.0218; 176.0267; 249.1606 | 267.0654 |
| Chlorogenic acid | (M-H)- | 59.0113; 191.0546; 309.0604 | 353.0816 |
| Gossypetin | (M-H)- | 53.8175; 117.6379; 166.9970; 231.0219; 302.0171 | 317.0256 |
| 2',6'-Dihydroxy-4'- methoxydihydrochalcone | (M-H)- | 81.0345; 109.0294 | 135.0438 |
| Hymecromone | (M-H)- | 91.0552; 119.0508; 131.0495; 147.0433; 160.0149 | 175.0390 |
| Caffeic acid | (M-H)- | 137.0595 | 179.034 |
| 6-Gingerol | (M-H)- | 96.9577 | 293.1737 |
| 2-Hydroxybenzoic acid | (M+H)+ | 83.16; 65.0380; 68.9968; 85.0620; 93.0332; 99.0667; 111.0433; 120.9518 | 139.0396 |
| Ellagic acid | (M+H)+ | 285.0044 | 303.0103 |
| Isopimpinellin | (M+H)+ | 111.0054; 177.0837; 249.2490 | 247.0587 |
| Gemfibrozil | (M+H)+ | 93.0686; 121.1000; 167.1059; 195.0972; 205.1575 | 251.1626 |
| Sinapyl aldehyde | (M+H)+ | 69.0318; 111.0451; 149.0560; 191.0752 | 209.08004 |
| Farrerol | (M+H)+ | 231.0623; 283.0911 | 301.1054 |
| Hispidulin | (M+H)+ | 167.0294; 264.4961 | 301.0664 |
| 4',5,6,7-Tetramethoxyflavone | (M+H)+ | 247.097; 275.0928 | 343.1172 |
| Verbenone | (M+H)+ | 69.0715 | 151.11164 |
| Curcumenol | (M+H)+ | 193.1569 | 235.1648 |
| Scopoletin | (M+H)+ | 121.0272; 147.0396 | 193.0487 |

**S3 (a) and (b).** The intensity of bioactive compounds of kānuka subcritical water extract identified using LC-MS/MS QTOF-ESI

<https://docs.google.com/spreadsheets/d/e/2PACX-1vQ3wZ4ksePbGKIZPM3pKbuGzux-7lg5QiiFKZnQ49mLjNEhWpCpA08H_XGO3GR5qQ/pubhtml?gid=350291390&single=true>
